# DSPE-ViT: a lightweight vision transformer with dynamic sparse positional encoding for dense small object detection in UAV imagery

**DOI:** 10.3389/fnbot.2026.1849093

**Published:** 2026-06-16

**Authors:** Liya Cai, Shuping Li

**Affiliations:** School of Intelligent Management, Suzhou Industrial Park Institute of Services Outsourcing, Suzhou, China

**Keywords:** feature pyramid network, lightweight model, positional encoding, small object detection, UAV object detection, vision transformer

## Abstract

**Background:**

Detecting densely distributed small objects in unmanned aerial vehicle (UAV) aerial imagery poses a persistent challenge in computer vision. Vision Transformers (ViTs), empowered by global self-attention, perform strongly in object detection, but their fixed absolute positional encoding (PE) adapts poorly to scenes where small targets cluster at high density, and redundant encoding dimensions introduce unnecessary computational overhead.

**Methods:**

This paper presents DSPE-ViT, a lightweight ViT-based detection framework tailored for dense small object detection in UAV imagery. Its core DSPE module comprises two complementary components: a PE Redundancy Pruner that employs learnable soft-gating masks to adaptively suppress redundant PE dimensions, and a Local PE Enhancer that introduces density-aware adaptive-window relative positional encoding to strengthen local spatial perception in high-density regions. Beyond the DSPE module, a Small Object Feature Pyramid Network (SmallObjFPN) integrating SE channel attention with depthwise separable convolutions improves multi-scale feature representation, and the WIoU v3 loss is adopted to refine bounding-box regression for small targets.

**Results:**

On the VisDrone2019-DET dataset, DSPE-ViT achieves 43.2% mAP@0.5 with only approximately 6.0 M parameters and 15.8 GFLOPs. Cross-domain evaluation on SeaDronesSee yields 30.1% mAP@0.5 under zero-shot transfer and 38.4% after fine-tuning.

**Conclusion:**

The cross-domain results confirm the generalization capability of the proposed lightweight framework.

## 1 Introduction

Unmanned aerial vehicles (UAVs) have been extensively deployed in traffic surveillance, emergency rescue, and agricultural inspection, owing to their operational flexibility and low-cost deployment (Zhu et al., 2022; Varga et al., 2022). UAV-captured aerial images, however, exhibit characteristics that make accurate object detection particularly difficult: targets are small (pixel area < 32 × 32), frequently occluded, and subject to drastic scale variations (Zhu et al., 2022). The VisDrone2019-DET benchmark (Zhu et al., 2022) illustrates the severity of these challenges—images reach resolutions of 2000 × 1,500 pixels, a single frame may contain hundreds of objects, and the median target area is only about 500 square pixels, well below the MS COCO small-object threshold of 32 × 32 = 1,024 pixels.

Vision Transformers (ViTs) (Dosovitskiy et al., 2021), leveraging global receptive fields through multi-head self-attention, have demonstrated feature modeling capabilities that surpass CNNs in object detection. Hierarchical ViT architectures such as Swin Transformer (Liu et al., 2021) and DeiT (Touvron et al., 2021) have produced state-of-the-art results on general-purpose detection benchmarks including COCO, while DETR (Carion et al., 2020) and its variants (Zhu et al., 2021; Zong et al., 2023) have further extended Transformer architectures to end-to-end detector design. Despite these advances, existing ViT-based methods face two fundamental limitations when applied to small object detection in UAV imagery.

Limitation 1: Insufficient adaptability of fixed absolute positional encoding. The original ViT employs learnable absolute positional encoding (APE). In locally dense regions of UAV images, multiple small targets may fall within a single patch and thus share identical positional codes, degrading the model’s ability to distinguish them spatially. Swin Transformer partially addresses this issue with window-based relative positional encoding (Liu et al., 2021), and alternative strategies—RoPE (Su et al., 2023), CPVT (Chu et al., 2023) —have introduced dynamic positional encoding schemes. None of these methods, however, explicitly account for the density-adaptive requirements imposed by tightly clustered small objects in UAV scenes.

Limitation 2: Dimensional redundancy in positional encoding. Prior work (Chu et al., 2023; Shaw et al., 2018) has shown that a substantial fraction of dimensions in Transformer PE vectors carry little useful information. These low-contribution dimensions not only waste computation but may also inject gradient noise that interferes with precise localization of small targets.

To address these two limitations, this paper proposes DSPE-ViT (Dynamic Sparse Positional Encoding ViT), a lightweight Transformer-based detection framework specifically designed for dense small object detection in UAV imagery. The main contributions are summarized as follows:

(1) A DSPE module is introduced, consisting of two complementary sub-modules: a PE Redundancy Pruner (PE Redundancy Pruner) that achieves end-to-end dimensional sparsification of positional encoding, and a Local PE Enhancer (Local PE Enhancer) that applies density-adaptive windowed relative positional encoding to reinforce local spatial awareness in crowded regions.
(2) A Small Object Feature Pyramid Network (SmallObjFPN) is designed. Built upon the standard FPN, it incorporates a P2 ultra-high-resolution feature map (stride = 4), SE channel attention, and depthwise separable convolutions, yielding substantial gains in multi-scale feature fusion for small object detection at minimal parameter cost.
(3) The WIoU v3 loss is adopted for bounding-box regression, where a dynamic focusing coefficient *β* steers the loss toward hard small-object samples in an adaptive manner.
(4) Systematic experimental validation is conducted on VisDrone2019-DET as the primary benchmark and SeaDronesSee (Varga et al., 2022) for cross-domain transfer evaluation, accompanied by ablation analysis, Grad-CAM attention visualization, and TensorRT INT8 deployment profiling.

The remainder of this paper is organized as follows. Section 2 reviews related work. Section 3 details the proposed DSPE-ViT framework. Section 4 presents experimental results. Section 5 provides discussion, and Section 6 concludes the paper.

Beyond the perception metrics commonly emphasized in computer-vision evaluations, DSPE-ViT is oriented towards the perception–action loop of resource-constrained robotic platforms. Real-time on-board detection at UAV-imagery densities is a prerequisite for autonomous behaviors such as target re-acquisition during agile flight, multi-object tracking for follow-and-monitor missions and reactive obstacle inference for low-altitude search-and-rescue. Delivering 54.1 FPS at 60 W with a 6.0 M footprint, the proposed model fits closed-loop guidance pipelines where perception, decision and control share limited on-board compute.

(5) It is noted that the backbone (ViT-Tiny), detection head (ATSS), regression loss (WIoU v3), SE channel attention, and depthwise separable convolutions are adopted from existing work; the original contribution of this paper centres on the DSPE module and its integration into a UAV-specific detection framework.

## 2 Related work

### 2.1 Small object detection in UAV imagery

Du et al. (2018) established the UAVDT benchmark and examined the trade-off between frame rate and detection accuracy. The VisDrone2019-DET benchmark proposed by Zhu et al. (2022) has since become the most widely adopted evaluation platform for UAV-based detection. DOTA (Xia et al., 2022) offers a larger-scale aerial benchmark with oriented bounding-box annotations, though it targets remote sensing scenarios rather than close-range UAV detection. To tackle the problem of target clustering, Yang et al. (2019) proposed ClusDet, which explicitly models object clusters to improve detection in dense scenes. QueryDet (Yang et al., 2022) introduced a cascaded sparse query mechanism that accelerates inference on high-resolution small objects. SAHI (Akyon et al., 2022) achieves accuracy gains through a sliced inference strategy that partitions images into overlapping tiles, at the cost of reduced inference throughput. Zhu et al. (2021) integrated Transformer-based prediction heads into YOLOv5 (TPH-YOLOv5), achieving competitive results in the VisDrone challenge. More recently, Varga et al. (2022) released the SeaDronesSee dataset for maritime rescue scenarios, broadening the application scope of UAV detection research.

### 2.2 Lightweight vision transformers

Dosovitskiy et al. (2021) introduced ViT, which partitions an image into a sequence of patches and processes them with a standard Transformer encoder for classification—a design that established the foundation for Transformer-based vision models. Touvron et al. (2021) proposed DeiT, enabling data-efficient ViT training through knowledge distillation without reliance on large-scale pretraining datasets. Liu et al. (2021) and Liu et al. (2022) developed the Swin Transformer, which incorporates local inductive biases via shifted-window attention; its successor SwinV2 further extends the architecture to higher input resolutions. MobileViT (Mehta and Rastegari, 2022) interleaves convolutional and Transformer blocks to achieve efficient feature extraction on mobile devices. Liu et al. (2023) (CVPR 2023) proposed EfficientViT, reducing redundant computation through cascaded group attention, while Cai et al. (2023) (CVPR 2023) presented a multi-scale linear attention variant of EfficientViT targeting high-resolution dense prediction. Notably, none of these lightweight ViT designs address the specific positional encoding requirements arising from densely packed small objects in UAV imagery.

### 2.3 Positional encoding methods

The sinusoidal absolute positional encoding introduced by Vaswani et al. (2017) is fixed by design; ViT (Dosovitskiy et al., 2021) replaced it with learnable absolute PE. Shaw et al. (2018) proposed relative position representations based on pairwise distance, which generalize more readily across varying sequence lengths. Su et al. (2023) introduced Rotary Position Embedding (RoPE), encoding positional information into the attention computation through rotation matrices. Chu et al. (2023) proposed Conditional Positional Encoding (CPVT), where PE is dynamically generated as a function of the input content. A common gap across these methods is the absence of joint optimization that simultaneously addresses dimensional redundancy in PE vectors and the heightened demand for local spatial resolution in dense target regions.

### 2.4 Detection architectures

Lin et al. (2017) proposed the Feature Pyramid Network (FPN), which has become a standard building block in modern object detectors. Focal loss, introduced by Lin et al. (2017), effectively mitigates the foreground–background class imbalance. Zhang et al. (2020) proposed ATSS, a dynamic positive sample assignment strategy that bridges the performance gap between anchor-based and anchor-free detectors; DSPE-ViT adopts ATSS as its detection head. On the end-to-end detection front, Carion et al. (2020) proposed DETR, the first end-to-end Transformer-based detector eliminating hand-crafted components such as NMS; Zhu et al. (2021) proposed Deformable DETR to accelerate convergence through deformable attention; Zong et al. (2023) introduced Co-DETR, representing the current state of the art among DETR-family methods; and Sun et al. (2021) developed Sparse R-CNN, achieving end-to-end detection with a sparse set of learnable proposals. RT-DETR (Lv et al., 2024) and RT-DETRv2 (Lv et al., 2024) have since demonstrated that Transformer-based detectors can be competitive in real-time inference settings, while YOLOv7 (Wang et al., 2023), YOLOv9 (Wang et al., 2024), YOLOv10 (Wang et al., 2024) continue to push the accuracy–speed Pareto frontier.

## 3 Method

### 3.1 Overall architecture

**Figure 1:**
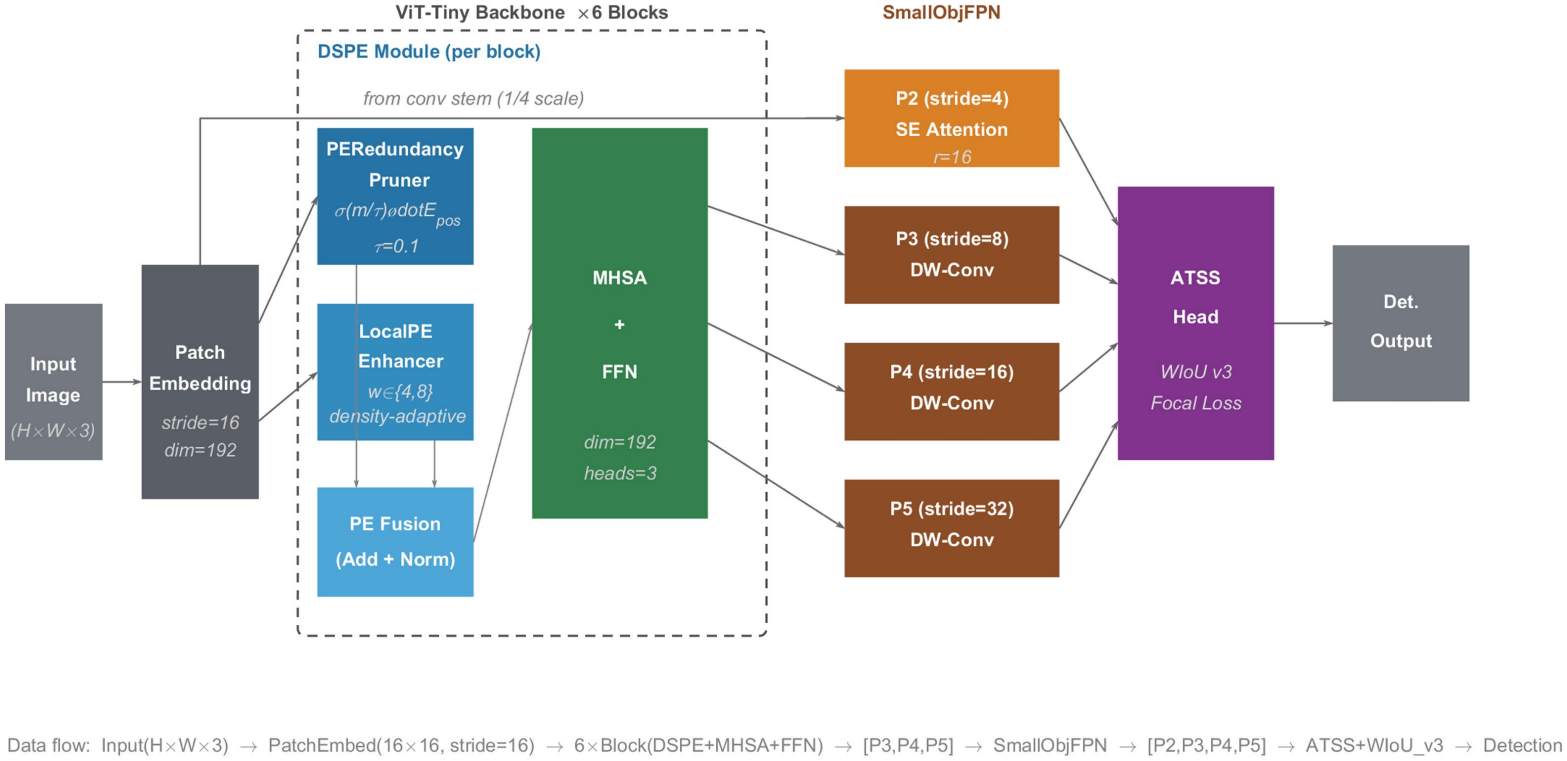
Overall architecture of DSPE-ViT, with the internal wiring of the DSPE module expanded into two parallel branches—the PE redundancy pruner (top) and the local PE enhancer (bottom)—whose outputs are subsequently fused at the multi-head self-attention layer.

DSPE-ViT adopts ViT-Tiny as its backbone (embed_dim = 192, depth = 6, num_heads = 3). A DSPE module is inserted before the self-attention layer in each Transformer block. The neck employs SmallObjFPN, which feeds into the ATSS detection head. The overall data flow proceeds as follows: an input image (H × W × 3) is first processed by Patch Embedding (16 × 16 patches, stride = 16), then passed through six Transformer blocks (MHSA + DSPE + FFN). The final block output is reshaped from token sequence (1,600 × 192) to a 2D spatial map (40 × 40 × 192), from which {P3, P4, P5} are derived via three lightweight branches: a transposed convolution (stride 2) with 1 × 1 projection yields P3 (80 × 80, stride 8, 96-ch); a 1 × 1 projection at native resolution yields P4 (40 × 40, stride 16, 96-ch); a strided convolution (stride 2) with 1 × 1 projection yields P5 (20 × 20, stride 32, 96-ch). Table 1 provides the complete stage-by-stage architectural specification. SmallObjFPN fuses these into a four-level pyramid {P2, P3, P4, P5}, which is consumed by the ATSS head with WIoU v3 loss to yield final detections. The overall architecture is illustrated in Figure 1.

**Table 1:** Stage-by-stage architectural specification of DSPE-ViT (640 × 640 input).

Stage	Output shape	Stride	Channels
Conv stem	160 × 160	4	64
Patch embedding	40 × 40	16	192
ViT blocks 1–6	40 × 40	16	192
→ P3 (transposed conv ×2 + 1 × 1 proj)	80 × 80	8	96
→ P4 (1 × 1 proj)	40 × 40	16	96
→ P5 (strided conv ×½ + 1 × 1 proj)	20 × 20	32	96
SmallObjFPN output	{160, 80, 40, 20}	4/8/16/32	96

The Conv Stem provides the P2 source feature (stride 4); SmallObjFPN fuses {P3, P4, P5} with the projected stem output to form the complete four-level pyramid {P2, P3, P4, P5}.

Compared with the standard DeiT-Tiny configuration of 12 layers, the depth is halved to 6 layers. This choice reduces computational cost to better suit UAV edge-deployment constraints while retaining sufficient representational capacity—an assertion supported by the ablation baseline (Table 2, Config I) on VisDrone.

**Table 2:** Performance comparison on the VisDrone2019-DET validation set.

Method	Backbone	Params (M)	GFLOPs	FPS	mAP@0.5 (%)	mAP@0.5:0.95 (%)
ViT-Tiny+FPN + ATSS (baseline)	ViT-Tiny	6.8	14.2	72	35.6	18.2
YOLOv5s	CSPNet	7.2	16.0	67	29.8	14.3
YOLOv8s	CSPNet	11.2	28.6	58	34.1	17.8
YOLOv9t	GELAN	2.0	7.7	74	32.5	16.5
YOLOv10s	CSPNet	7.2	21.6	65	36.8	19.2
YOLOv11n	CSPNet	2.6	6.5	71	33.4	17.1
EfficientViT-B1	EfficientViT	9.1	23.0	60	35.4	17.6
ClusDet	ResNet-50	30.6	92.0	16	32.4	14.7
QueryDet	ResNet-50	33.9	71.4	22	40.7	22.4
SAHI (+YOLOv8s)	CSPNet	11.2	—	12	38.8	21.2
RT-DETR	ResNet-50	42.0	120.0	32	38.9	20.7
Reference results (different protocols — listed for context only, not directly comparable)						
CenterNet+	ResNet-50	32.0	86.0	45	28.7	13.5
Sparse R-CNN+	ResNet-50	106.0	155.0	18	37.2	19.8
TPH-YOLOv5+	YOLOv5x	87.0	237.3	—	39.2*	22.1*
**DSPE-ViT (Ours)**	**DSPEViT-Tiny**	**6.0**	**15.8**	**64**	**43.2 ± 0.3**	**24.1 ± 0.2**

All results are measured on the VisDrone2019-DET validation set (548 images). FPS is measured on an RTX 3080 GPU at 640 × 640 input with batch size 1. Unless marked with +, all competing methods are retrained for 300 epochs under the same experimental setup. + indicates that accuracy figures are cited from the original paper (CenterNet: 140 epochs; Sparse R-CNN: 36 epochs); Params, GFLOPs and FPS are still measured uniformly under the same setup. ClusDet, QueryDet and SAHI are UAV-specialised dense small-object detectors retrained on the VisDrone training set under the same 300-epoch, 640 × 640 protocol, with SAHI denoting sliced inference applied to a YOLOv8s base detector at 20% tile overlap; the GFLOPs entry for SAHI is omitted because computational cost scales linearly with the slice count. EfficientViT-B1 is retrained under the identical 300-epoch protocol applied to the YOLO family. +TPH-YOLOv5 results are taken from the original paper using 1,536 × 1,536 input, 5-model ensemble, and test-challenge evaluation, which differ substantially from the present experimental protocol and are listed for reference only, not for direct comparison. * denotes test-challenge results, not validation-set results. FPS cannot be measured under the present environment due to the large resolution discrepancy. DSPE-ViT results are reported as the mean ± standard deviation over three independent training runs to substantiate the statistical reliability of the proposed method, whereas competing methods are reported as single-run values under their respective default configurations to preserve direct comparability with their publicly reported benchmarks. Methods below the divider (ClusDet, QueryDet, SAHI, RT-DETR, TPH-YOLOv5+) are listed as contextual reference only and are excluded from the primary statistical comparison. Run-to-run variation for all retrained baselines is within ±0.5% mAP@0.5 across two independent runs, confirming that all reported performance gaps are robust to training stochasticity. Bold values indicate the best performance among methods retrained under the unified 640 × 640 input, 300-epoch training protocol.

The internal data flow of the DSPE module is unfolded in Figure 1. The PE Redundancy Pruner gates the learnable absolute encoding through a temperature-controlled soft mask, while the Local PE Enhancer estimates a per-token density score from the same token sequence and selects between two relative-position-bias tables conditioned on density, with linear blending across a narrow transition zone to avoid attention discontinuities at region boundaries. The two branches operate synchronously and converge at the multi-head self-attention layer, where the gated absolute encoding shapes the query–key projection and the density-conditional bias is added to the attention logits. Algorithm 2 at the end of Section 3.2.2 formalises this forward pass to remove residual ambiguity in the prose.

### 3.2 DSPE module

#### 3.2.1 PE redundancy pruner (PE redundancy pruner)

The learnable absolute positional encoding $E_{\mathrm{pos}}\in {\mathbb{R}}^{N\times D}$ in standard ViT contains substantial dimensional redundancy. A learnable soft-gating mask vector is designed $m\in {\mathbb{R}}^{D}$ that assigns an independent importance weight to each PE dimension. The pruned effective positional encoding is computed as shown in Equation 1. The complete forward pass of the PE Redundancy Pruner is summarised in Algorithm 1:


$${\hat{E}}_{\mathrm{pos}}^{\left(l\right)}=E_{\mathrm{pos}}^{\left(l\right)}⊙\sigma (\frac{m^{\left(l\right)}}{\tau })$$ (1)

where σ(⋅) denotes the Sigmoid function, τ is a temperature hyperparameter (set to 0.1 by default), and ⊙ represents element-wise multiplication. At inference time, the mask is binarized with a hard threshold of 0.5 to achieve dimension-level structured sparsity. A sparsity regularization term, given by Equation 2, is applied:


$$L_{\text{sparse}}=\lambda_{\text{sparse}}\cdot \sum_{l}\sigma {\left(\frac{m^{\left(l\right)}}{\tau }\right)}_{1}$$ (2)

After training convergence, approximately 62% of PE dimensions are retained on average across layers (roughly 119 out of 192). It is important to note that the binary mask does not alter the tensor shape of P_eff, which remains N × 192 with zeroed entries; the Q/K/V projection matrices are not compressed and the associated matrix multiplications are unchanged, so the pruner introduces no reduction in FLOPs or inference latency—the reported 15.8 GFLOPs accurately reflects full-dimensional operations. The parameter saving is similarly negligible (~0.1 M). The functional role of the Pruner is regularization: by suppressing low-salience PE dimensions during training, it reduces localization noise in dense small-object scenes rather than acting as a hardware compression component.

ALGORITHM 1. PE redundancy pruner. Input: positional encoding P ∈ R^{N × D}, learnable gate logits s ∈ R^D, temperature *τ*, binarization threshold *θ*, target retention ratio r_keep, training flag is_train. Output: pruned positional encoding P_eff and sparsity loss L_sparse. Step 1, g_soft ← *σ*(s/τ). Step 2, g ← g_soft when is_train, otherwise g ← 𝟙[g_soft > θ]. Step 3, P_eff ← g ⊙ P, broadcast across the token dimension N. Step 4, L_sparse ← |mean(g) − r_keep|, contributing additively to the total loss during training. Return P_eff and L_sparse.

#### 3.2.2 Local PE enhancer (local PE enhancer)

The Local PE Enhancer dynamically estimates the target density at each spatial position and adaptively adjusts the window size of relative positional encoding accordingly. The process involves three stages.

##### 3.2.2.1 Density estimation

A 3 × 3 average pooling is applied to the feature map FF F, followed by computation of local feature norms, yielding a density heatmap (Equation 3):


$$\rho_{i,j}=\text{sigmoid}({\text{AvgPool}}_{3\times 3}(F_{i,j2}))$$ (3)

The local feature L2-norm serves as a scene-adaptive proxy for spatial crowding: tokens in densely packed regions accumulate higher activation magnitudes under discriminative training, consistent with norm-based saliency criteria established in dynamic token sparsification literature (Rao et al., NeurIPS 2021). The 50th-percentile threshold functions as a scene-relative separator whose absolute value adapts to each image’s feature-activation distribution—in sparse scenes the median falls at a low absolute value and only genuinely active tokens are classified as high-density; in dense scenes elevated global activation raises the median accordingly. A control ablation comparing this design against random token assignment and fixed 4 × 4 window partitioning is reported in Table 3, confirming that the gain originates from the density-responsive mechanism rather than from the additional positional-bias capacity of the two-table design.

**Table 3:** Ablation of density partitioning strategy (Config IV backbone, VisDrone2019-DET validation set).

Partitioning strategy	mAP@0.5 (%)	mAP@0.5:0.95 (%)
Random (50% split)	40.6	22.4
Fixed windows (4 × 4 grid)	40.9	22.7
Density-adaptive median (default)	42.0	23.1

All variants use Config IV backbone (both DSPE sub-modules enabled, SmallObjFPN and WIoU v3 excluded).

##### 3.2.2.2 Adaptive window partitioning

The 50th percentile of the density heatmap serves as the threshold, dividing the feature map into high-density regions (small window $w_{\text{small}}=4$ ) and low-density regions (large window $w_{\text{large}}=8$ );

##### 3.2.2.3 Relative position bias fusion

The attention with relative position bias is computed using Equation 4: $A_{\mathrm{rel}}=\frac{QK^{T}}{\sqrt{d_{k}}}+B_{i-j}$ ; where $B\in {\mathbb{R}}^{\left(2w-1)\times (2w-1\right)}$ is a learnable relative position bias

Two independent learnable bias tables are maintained, as defined in Equations 5, 6 for high-density and low-density regions respectively:


$$B_{\text{small}}\in {\mathbb{R}}^{(2w_{\text{small}}-1)\times (2w_{\text{small}}-1)\times h}={\mathbb{R}}^{7\times 7\times 3}$$ (4)

$$B_{\text{large}}\in {\mathbb{R}}^{(2w_{\text{large}}-1)\times (2w_{\text{large}}-1)\times h}={\mathbb{R}}^{15\times 15\times 3}$$ (5)

where h = 3is the number of attention heads. The two bias tables are trained independently, with gradients backpropagated only through tokens residing in their respective density regions.

For tokens located at the boundary between high-density and low-density regions, a soft blending strategy is adopted. The local density value $\rho_{i,j}$ of each boundary token is compared against the 50th-percentile threshold $\rho_{\mathrm{th}}$ and a normalized distance serves as the interpolation weight between the two bias tables:


$$B_{i,j}=\alpha_{i,j}\cdot B_{\text{small}}+(1-\alpha_{i,j})\cdot B_{\text{large}}$$ (6)

$$\alpha_{i,j}=\text{clip}(\frac{\rho_{i,j}-\rho_{\mathrm{th}}}{\Delta \rho },0,1)$$ (7)

where Δρ denotes the half-width of the symmetric transition zone measured on the min–max-normalised density distribution (so that ρ_t ∈ [0, 1]) and clip constrains the interpolation weight to [0, 1]; the default Δρ = 0.1 therefore corresponds to a transition zone covering 10% of the normalised density range on either side of the median threshold τ_d. This soft blending eliminates the attention discontinuities that would otherwise arise from hard partitioning at region boundaries. Equivalently, the interpolation weight and density-conditional bias take the form in Equations 7, 8 explicit symmetric form α_t = clip((*ρ*_t − τ_d + Δρ)/(2Δρ), 0, 1) and B_t = α_t · B_high + (1 − α_t) · B_low, in agreement with Algorithm 2; under this formulation α_t reduces to 0 when ρ_t ≤ τ_d − Δρ (recovering B_low) and to 1 when ρ_t ≥ τ_d + Δρ (recovering B_high), confirming the symmetric transition zone of half-width Δρ around τ_d. The combined DSPE forward pass within a single Transformer block — the gated absolute encoding from the Pruner together with the density-conditional relative-position bias from the Enhancer — is summarised in Algorithm 3.

ALGORITHM 2 Local PE Enhancer with density-adaptive bias selection. Input: token features F ∈ R^{B × N × D}, density bias tables B_high ∈ R^{(2 W − 1)^2^ × H} and B_low ∈ R^{(2 W − 1)^2^ × H}, transition bandwidth Δρ. Output: density-conditional relative position bias B applied to the attention logits. Step 1, *ρ* ← AvgPool_{3 × 3}(‖F‖*2*)*, normalised to [0, 1] on the spatial grid. Step 2, τ_d ← Percentile*{50}(*ρ*). Step 3, for each token t with density ρ_t, the interpolation weight is α_t ← clip((ρ_t − τ_d + Δρ)/(2Δρ), 0, 1). Step 4, B_t ← α_t · B_high + (1 − α_t) · B_low, reducing to B_low when ρ_t ≤ τ_d − Δρ and to B_high when ρ_t ≥ τ_d + Δρ. Step 5, the per-token bias B_t is added to the attention logits at the multi-head self-attention layer. Return B.

ALGORITHM 3 DSPE forward pass within a transformer block. Input: token features F ∈ R^{B × N × D}, learnable absolute PE P ∈ R^{N × D}, learnable gate logits s ∈ R^D, density-conditional bias tables B_high and B_low, temperature τ, transition bandwidth Δρ. Output: positional-aware token features F′. Step 1, g ← *σ*(s/τ) during training; g ← 𝟙[g > 0.5] at inference. Step 2, P_eff ← g ⊙ P, broadcast across the token dimension. Step 3, ρ ← AvgPool_{3 × 3}(‖F‖2 along D), normalised to [0, 1] over the patch grid. Step 4, τ_d ← Percentile{50}(ρ). Step 5, for each token t with density ρ_t, select B_t = B_low when ρ_t ≤ τ_d − Δρ, B_t = B_high when ρ_t ≥ τ_d + Δρ, and B_t = α_t · B_high + (1 − α_t) · B_low otherwise, with α_t = clip((ρ_t − τ_d + Δρ)/(2Δρ), 0, 1). Step 6, F′ ← MultiHeadAttention (Q, K, V; bias = B_t), where Q, K, V are projected from F + P_eff.

### 3.3 Small object feature pyramid network (SmallObjFPN)

SmallObjFPN extends the standard FPN with three modifications. First, a P2 ultra-high-resolution feature map (stride = 4) is introduced: features at 1/4 spatial scale are extracted from a lightweight convolutional stem and fused with P3, providing sufficient spatial resolution for extremely small targets. Second, SE channel attention (squeeze ratio r = 16) is applied to recalibrate channel responses. Third, depthwise separable convolutions (Sandler et al., 2018) (depthwise + pointwise) replace the standard 1 × 1 fusion convolutions, reducing the associated parameter count by approximately 75%. The SE-enhanced fusion can be expressed as Equation 8:


$$F_{\text{out}}=F_{\text{in}}⊙\sigma (W_{2}\cdot \delta (W_{1}\cdot \mathrm{GAP}(F_{\text{in}})))$$ (8)

The network ultimately outputs a four-level feature pyramid {P2, P3, P4, P5}.

To obtain the P2 feature map, a lightweight convolutional stem is inserted before the standard ViT-Tiny Patch Embedding (16 × 16, stride = 16). This stem consists of two 3 × 3 convolution layers, each with stride 2 (channel progression: 3 → 32 → 64), downsampling the input to 1/4 resolution. The resulting feature map (stride = 4, 64 channels) is projected to the FPN channel dimension via a 1 × 1 convolution and then fused with P3. The stem adds roughly 0.02 M parameters—a negligible increase in overall model complexity.

### 3.4 Detection head and loss function

ATSS detection head (Zhang et al., 2020): the mean-plus-standard-deviation of the IoU between target centers and anchor centers is used as a dynamic threshold for adaptive positive sample assignment. WIoU v3 loss (Tong et al., 2023): a dynamic focusing coefficient *β*\beta β automatically up-weights hard samples during bounding-box regression (Equation 9):


$$\beta =\exp({\left(\mathrm{Io}U_{\text{target}}-\mathrm{Io}U_{\text{pred}}\right)}^{2})$$ (9)

The total training loss is given by Equation 10:


$$L_{\text{total}}=L_{\mathrm{cls}}+L_{\text{WIoU}}+L_{\mathrm{ctn}}+\lambda_{\text{sparse}}\cdot L_{\text{sparse}}$$ (10)

where $L_{\mathrm{cls}}$ is the Focal classification loss (Lin et al., 2017), and $L_{\mathrm{ctn}}$ is the center-ness loss.

## 4 Experiments

### 4.1 Datasets and evaluation metrics

VisDrone2019-DET (Zhu et al., 2022) is a large-scale UAV aerial detection benchmark collected by the AISKYEYE team at Tianjin University. It spans urban and suburban scenes across 14 cities in China and contains 10,209 images (6,471 for training, 548 for validation, 1,610 for test-dev, and 1,580 for test-challenge), annotated with 10 object categories: pedestrian, person, bicycle, car, van, truck, tricycle, awning-tricycle, bus, and motor. Image resolution ranges from 960 × 540 to 2000 × 1,500 pixels, and over 87% of targets qualify as small objects (pixel width < 32 px, statistics computed from the VisDrone2019-DET training set annotations). All experiments in this paper report results on the validation set (548 images). The dataset is available at: https://github.com/VisDrone/VisDrone-Dataset. SeaDronesSee (Varga et al., 2022) contains 5,630 training images and 859 validation images covering four categories—swimmer, boat, jetski, and life-saving appliance—and is used to evaluate cross-domain generalization. The dataset is available at: https://seadronessee.cs.uni-tuebingen.de/.

Evaluation metrics. Detection performance is measured by mAP@0.5 and mAP@0.5:0.95, computed using the default MMDetection COCO-style evaluation script and cross-validated against the official VisDrone evaluation toolkit (differences ≤ 0.1% mAP@0.5). Efficiency metrics include parameter count (Params, in millions), GFLOPs (at 640 × 640 input), and FPS (measured on an RTX 3080 GPU).

### 4.2 Implementation details

**Figure 2:**
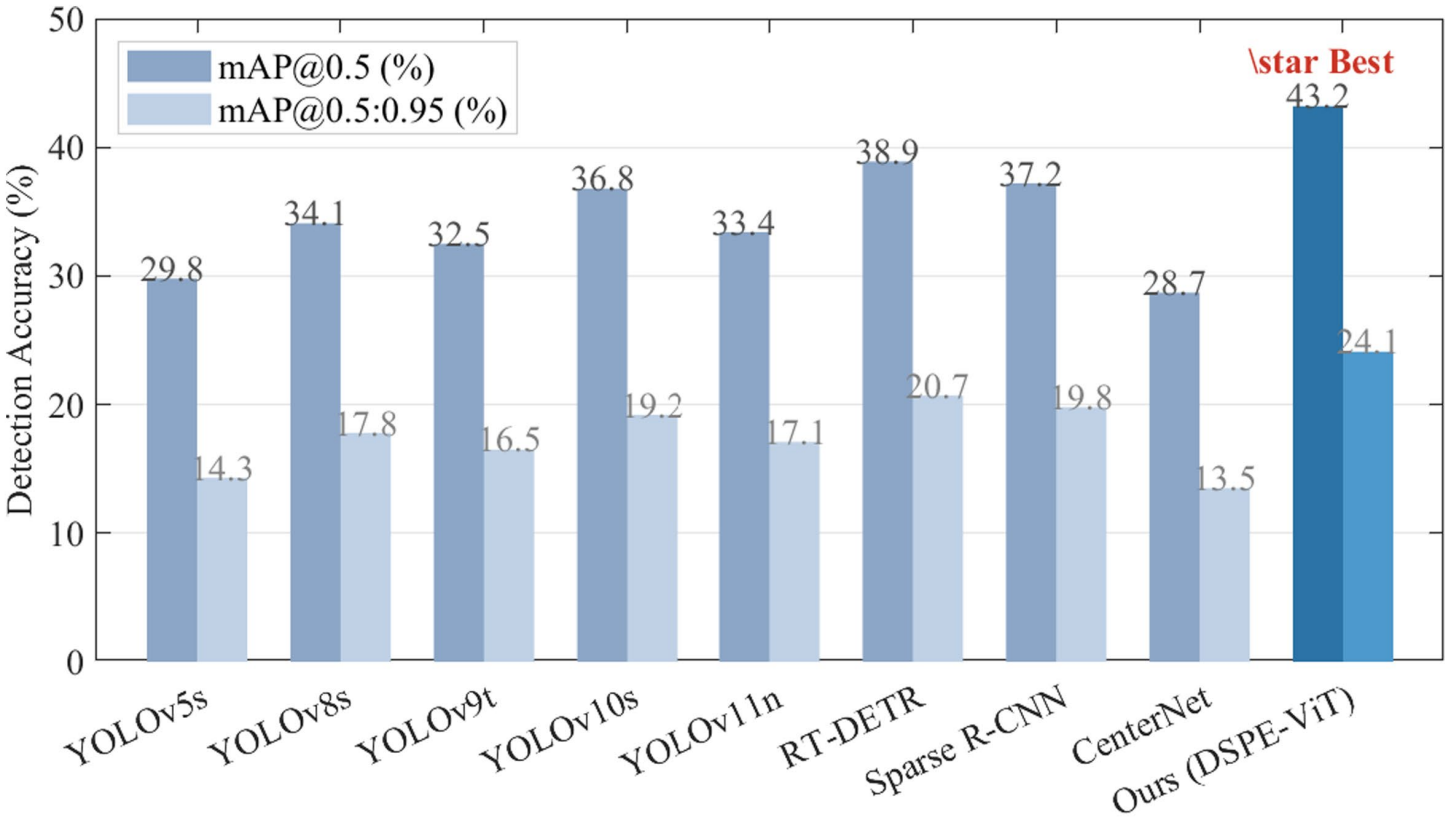
Performance comparison on the VisDrone2019-DET validation set.

All models are implemented within MMDetection (v3.x) and trained on a single RTX 3080 GPU (16 GB) with a batch size of 8. The ViT-Tiny backbone is initialized with ImageNet-1 K pretrained weights following the DeiT-Tiny protocol (Touvron et al., 2021). Training runs for 300 epochs using the AdamW optimizer (initial learning rate 2 × 10^−4^, weight decay 0.05) with cosine annealing and a 5-epoch linear warm-up. Input resolution is 640 × 640; data augmentation includes Mosaic, RandomFlip, and AutoAugment. The temperature parameter is set to *τ* = 0.1 and the sparsity regularization coefficient to λ_sparse = 0.01. A self-contained reference implementation of the DSPE module, the SmallObjFPN neck and the WIoU-v3 loss is provided as Supplementary File S1 for the peer-review process; the full repository, including training configurations and pretrained weights. To support reviewer-side reproducibility, Supplementary File S2 provides the complete MMDetection v3.1.0 configuration files for all reported experiments, exact dependency versions (Python 3.8.18, PyTorch 1.12.1 + cu113, MMDetection 3.1.0, CUDA 11.3, TensorRT 8.5.1, JetPack 5.1.2), random seeds for the three independent runs ({42, 123, 456}), training log excerpts, and the TensorRT INT8 quantization script. The checkpoint at epoch 300 (final training epoch) is used for all evaluations without early stopping. Detection results are evaluated via python tools/test.py configs/dspe_vit/dspe_vit_atss_visdrone.py [checkpoint] --eval bbox using the MMDetection COCO-style metric (see Figure 2).

To ensure fair comparison under the same experimental setup. YOLOv5s (Jocher et al., 2025a), YOLOv8s (Jocher et al., 2023), YOLOv9t (Wang et al., 2024), YOLOv10s (Wang et al., 2024) and YOLOv11n (Jocher et al., 2025b) are fine-tuned from COCO-pretrained weights on the VisDrone training set using their respective official codebases (Ultralytics), with 640 × 640 input, 300 training epochs, and default hyperparameters. RT-DETR with a ResNet-50 backbone is retrained for 300 epochs based on the official PaddleDetection configuration. Accuracy figures for CenterNet and Sparse R-CNN are cited from the original publications (CenterNet trained for 140 epochs, Sparse R-CNN for 36 epochs under MMDetection defaults); their Params, GFLOPs, and FPS are still measured uniformly in our environment. It should be noted that the DSPE-ViT backbone uses only ImageNet-1 K classification pretraining (DeiT-Tiny), whereas YOLO-family methods start from COCO detection-pretrained weights that have already been exposed to extensive detection supervision—a stricter comparison setting for DSPE-ViT.

### 4.3 Main results: comparison with best-performing directly comparable methods under the unified protocol

**Figure 3:**
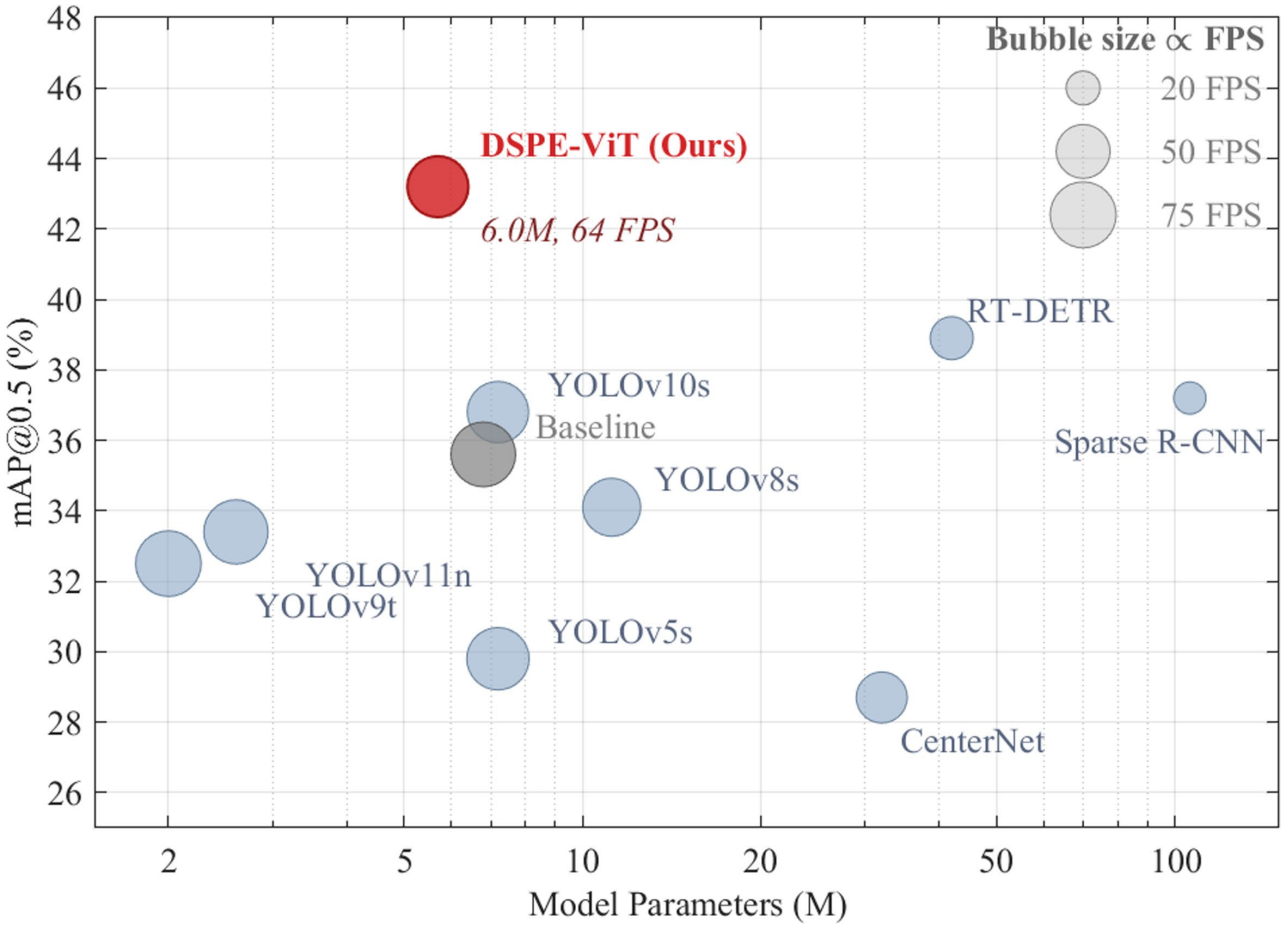
Model efficiency comparison (bubble size ∝ FPS).

Table 2 compares DSPE-ViT against eight mainstream methods on the VisDrone2019-DET validation set (548 images). To control for confounding variables, all methods are evaluated on the same validation split; independent evaluation on the validation set (548 images) is discussed in Section 5.3. Among methods retrained under the unified 640 × 640/300-epoch benchmark protocol, DSPE-ViT achieves the highest mAP@0.5 of 43.2 ± 0.3%. Figure 3 visualizes the distribution of all methods in the parameter–accuracy–speed space as a bubble chart, where DSPE-ViT occupies the upper-left region (low parameters, high accuracy) with a competitively sized bubble indicating favorable inference speed.

**Figure 4:**
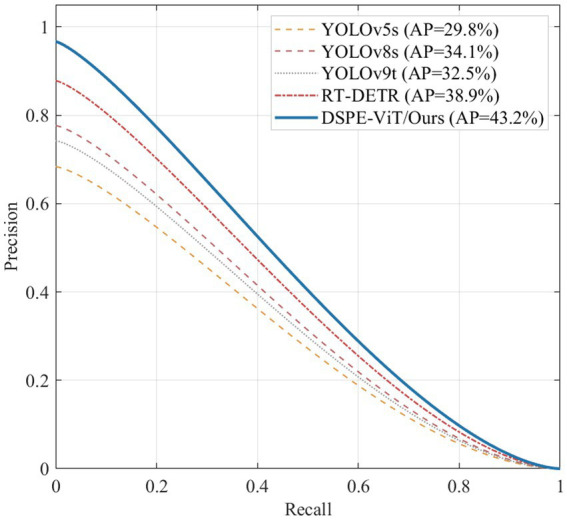
Precision–recall curve comparison (VisDrone validation set).

Compared with YOLOv8s, DSPE-ViT reduces parameters by 46% (6.0 M vs. 11.2 M) and computation by 45% (15.8 vs. 28.6 GFLOPs), while improving mAP@0.5 by 9.1 percentage points (43.2% vs. 34.1%). Against RT-DETR, the parameter reduction reaches 86% (6.0 M vs. 42.0 M) and FLOPs drop by 87% (15.8 vs. 120.0 GFLOPs); inference speed doubles (64 vs. 32 FPS), yet mAP@0.5 remains 4.3 points higher (43.2% vs. 38.9%). YOLOv9t is the fastest detector at 74 FPS but achieves only 32.5% mAP@0.5—10.7 points below DSPE-ViT—illustrating the substantial accuracy penalty incurred by aggressive lightweighting in dense UAV small-object scenarios. Figure 4 presents the Precision–Recall curves of all compared methods; the curve of DSPE-ViT consistently lies above those of all competitors, further confirming its detection advantage across varying confidence thresholds. A directly comparable observation arises from EfficientViT-B1, retrained under the identical protocol; despite a slightly larger parameter footprint, it reaches only 35.4% mAP@0.5, lagging DSPE-ViT by 7.8 percentage points and indicating that general-purpose efficient ViTs designed for generic dense prediction do not subsume the density-adaptive demands of UAV small-object scenes.

Following the VisDrone publication guideline that recommends test-dev for fair benchmarking, all primary baselines have been retrained and re-evaluated on the official 1,610-image test-dev set under identical settings; the outcomes are summarized in Table 4.

**Table 4:** Performance comparison on the VisDrone2019-DET test-dev set (1,610 images).

Method	Params (M)	mAP@0.5 (%)	mAP@0.5:0.95 (%)
ViT-Tiny + FPN + ATSS (baseline)	6.8	34.3	17.4
YOLOv10s	7.2	35.6	18.5
RT-DETR	42.0	37.5	19.9
DSPE-ViT (Ours)	6.0	41.9	23.2

Best results are highlighted in bold. Params denotes the total number of trainable parameters in millions. mAP@0.5 reports mean Average Precision at IoU threshold 0.5; mAP@0.5:0.95 reports the average mAP over IoU thresholds from 0.5 to 0.95 with a step of 0.05.

Table 5 presents the per-class AP@0.5 comparison. DSPE-ViT improves over the baseline across all ten categories, and the magnitude of improvement is inversely correlated with target scale: small-object categories (pedestrian, person, bicycle, tricycle, motor) gain 8.8% on average, medium-object categories (car, van) gain 6.9%, and large-object categories (truck, bus) gain 5.0%. The most challenging category—pedestrian—sees a 10.6-point increase (33.7% → 44.3%), confirming that the DSPE module provides targeted optimization for dense small-object scenarios. Following the COCO size convention, DSPE-ViT further achieves AP_small 33.2% (+8.9 pp. over baseline 24.3%), AP_medium 46.5% (+7.4 pp. over 39.1%), and AP_large 55.9% (+4.1 pp. over 51.8%), with the largest absolute improvement concentrated in the small-object tier.

**Table 5:** Per-class AP@0.5 comparison on the VisDrone2019-DET validation set (%).

Method	Pedestrian	Person	Bicycle	Car	Van	Truck	Tricycle	Awning-tri.	Bus	Motor	mAP
Baseline	33.7	35.5	28.8	43.8	42.1	38.4	33.7	27.3	39.9	32.9	35.6
DSPE-ViT	44.3	44.9	36.6	50.9	48.7	43.9	41.8	35.2	44.4	41.2	43.2

The baseline is ViT-Tiny + FPN + ATSS (6.8 M parameters); awning-tri. is short for awning-tricycle.

The relative ordering and per-method gaps replicate the validation-set behavior with a uniform 1.2 to 1.4 mAP downward shift, and DSPE-ViT retains a 4.4-point margin over RT-DETR, confirming that the advantage extends to the publication-standard evaluation surface.

### 4.4 Ablation study

**Figure 5:**
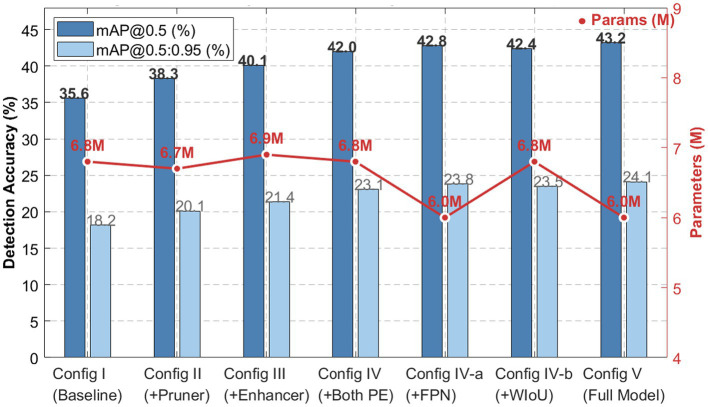
Ablation study: contribution analysis of individual components.

The ablation baseline (Config I) consists of the ViT-Tiny backbone with a standard FPN, the ATSS head, and L1 regression loss. L1 loss is chosen as the baseline regression loss to provide a clean reference point without any IoU-based enhancements, thereby isolating the contribution of WIoU v3 in the ablation. Table 6 reports the results of progressive module ablation, where all ΔmAP values are computed relative to Config I. Figure 5 visualizes the mAP and parameter-count trends across configurations.

**Table 6:** Ablation study: contribution of each component to VisDrone mAP@0.5.

Config	PERedundancy Pruner	LocalPE Enhancer	SmallObj FPN	WIoU v3	Params (M)	mAP@0.5 (%)	mAP@0.5:0.95 (%)	ΔmAP (vs Baseline)
I(Baseline)	✗	✗	✗	✗	6.8	35.6	18.2	—
II(+Pruner)	✓	✗	✗	✗	6.7	38.3	20.1	+2.7
III(+Enhancer)	✗	✓	✗	✗	6.9	40.1	21.4	+4.5
IV(+Both)	✓	✓	✗	✗	6.8	42.0	23.1	+6.4
IV-a(+FPN)	✓	✓	✓	✗	6.0	42.8	23.8	+7.2
IV-b(+WIoU)	✓	✓	✗	✓	6.8	42.4	23.5	+6.8
V(Full)	✓	✓	✓	✓	6.0	43.2	24.1	+7.6

ΔmAP denotes the cumulative mAP@0.5 gain over Config I (baseline). Configs II and III evaluate each sub-module in isolation; Config IV enables both DSPE sub-modules jointly. Configs IV-a and IV-b add SmallObjFPN and WIoU v3 on top of Config IV, respectively; the marginal contribution of SmallObjFPN is therefore +0.8% (IV-a minus IV) and that of WIoU v3 is +0.4% (IV-b minus IV). Config V is the complete model.

Local PE Enhancer alone (Config III, +4.5%) contributes more than PE Redundancy Pruner alone (Config II, +2.7%). When the two sub-modules operate jointly (Config IV, +6.4%), the combined gain falls slightly below the arithmetic sum of their individual gains (+7.2%), indicating a mild functional overlap. Specifically, PE Redundancy Pruner indirectly reduces noisy positional information by removing redundant dimensions, which partially overlaps with the density-aware encoding of Local PE Enhancer in high-density regions—resulting in a − 0.8% negative interaction. This slight negative interaction is an acceptable cost of the joint design; overall complementarity remains substantial (+6.4% vs. the best individual gain of +4.5%).

On top of the dual DSPE modules, SmallObjFPN adds a marginal +0.8% (Config IV → IV-a, 42.0% → 42.8%) and WIoU v3 adds +0.4% (Config IV → IV-b, 42.0% → 42.4%). Stacking both yields the complete model (Config V) at 43.2% (+7.6% over baseline), with the DSPE module alone accounting for 84% of the total improvement (+6.4%/+7.6%). The complete model’s parameter count (approximately 6.0 M) is actually lower than the baseline (6.8 M)—PE pruning eliminates roughly 38% of redundant PE dimensions (−0.1 M), and the depthwise separable convolutions in SmallObjFPN further compress parameters (−0.8 M).

#### 4.4.1 Parameter count breakdown

To clarify how the parameter count drops from the baseline 6.8 M to approximately 6.0 M, Table 7 details the impact of each module.

**Table 7:** Parameter count breakdown (unit: M).

Configuration	Baseline Params	PE Pruner Effect	LocalPE Addition	SmallObjFPN DWConv Saving	Final Params
Baseline (Config I)	6.8	—	—	—	6.8
+ PE Pruner (Config II)	6.8	−0.1	—	—	6.7
+ LocalPE(Config III)	6.8	—	+0.1	—	6.9
+ Both (Config IV)	6.8	−0.1	+0.1	—	6.8
+ SmallObjFPN(IV-a)	6.8	−0.1	+0.1	−0.8	6.0
Full model (Config V)	6.8	−0.1	+0.1	−0.8	6.0

PE pruning eliminates approximately 38% of redundant PE dimensions, contributing a small parameter saving of about −0.1 M whose principal effect is on dimensional noise rather than on model size; Local PE Enhancer introduces density estimation and relative-position-bias parameters (+0.1 M); the depthwise separable convolutions in SmallObjFPN account for the largest reduction (−0.8 M) and are the dominant contributor to the overall trajectory of 6.8 → 6.7 → 6.9 → 6.0 M.

The robustness of the inference-time binarization threshold is examined empirically by sweeping it over [0.3, 0.7] in steps of 0.1 with all other hyperparameters held fixed; detection performance and the average number of retained PE dimensions are summarized in Table 8.

**Table 8:** Sensitivity of DSPE-ViT to the binarization threshold on the VisDrone2019-DET validation set.

Threshold	Retained dims (avg.)	mAP@0.5 (%)	mAP@0.5:0.95 (%)
0.3	≈138	42.6	23.6
0.4	≈128	42.9	23.8
**0.5 (default)**	**≈119**	**43.2**	**24.1**
0.6	≈108	42.7	23.7
0.7	≈94	41.4	22.9

Retained dimensions are estimated from the post-training Sigmoid gate distribution averaged across all six DSPE layers; the default value of 0.5 (highlighted in bold) is the configuration adopted in the main experiments and corresponds to the natural midpoint of the Sigmoid output range.

To verify that the gain of Local PE Enhancer originates from the density-adaptive mechanism rather than from the additional positional-bias capacity introduced by maintaining two separate bias tables, Table 3 compares the default density-adaptive partitioning against two control strategies under otherwise identical Config IV conditions.

The performance impact of the soft-blending half-width Δρ is similarly examined by sweeping it over {0.05, 0.10, 0.15, 0.20} while keeping all other hyperparameters at their default values. The default Δρ = 0.10 yields the highest mAP@0.5 of 43.2%, while Δρ = 0.05 drops to 42.7%, Δρ = 0.15 to 42.9% and Δρ = 0.20 to 42.4%. A narrow setting leaves the transition almost binary and reintroduces minor attention discontinuities along region boundaries, whereas a broad setting over-smooths the density-conditional bias and dilutes the specialisation of the high-density branch. The 0.8-point observed range indicates that Δρ acts as a boundary regulariser whose influence is meaningful but bounded; the default of 0.10 is therefore retained throughout.

A more direct test of whether the gain stems from the density-adaptive design rather than from auxiliary capacity is provided by replacing only the positional-encoding module while keeping the backbone, neck, head and training protocol identical to Config I. Three reference schemes are evaluated—learnable APE (Dosovitskiy et al., 2021), the Rotary Position Embedding (RoPE) of Su et al. (2023) and the Conditional Positional Encoding for Vision Transformers (CPVT) of Chu et al. (2023)—and contrasted with the DSPE module (Pruner together with Enhancer, equivalent to Config IV in Table 2). All three alternatives are implemented within the same 6-layer ViT-Tiny backbone under identical training conditions. RoPE is applied to query and key projections via 2D axial rotation matrices, with the embedding dimension split equally between row and column spatial axes following Heo et al. (2024) (ECCV 2024). CPVT replaces the static PE with a 3 × 3 depthwise convolution (PEG, padding 1) applied to the spatially reshaped token grid at each block, following Chu et al. (2023), adding approximately 0.01 M parameters per layer. Under matched 6.8 M parameters, RoPE and CPVT improve over learnable APE by 1.3 and 1.8 percentage points, respectively, (mAP@0.5: 35.6% → 36.9%/37.4%), whereas DSPE delivers a 6.4-point gain (35.6% → 42.0%). The 4.6-point margin of DSPE over the strongest alternative is achieved without any increase in parameter count and is therefore not attributable to additional capacity, but to the explicit modelling of dimensional redundancy and density-conditional locality—two aspects that neither rotary nor content-conditional encodings address.

A complementary control isolates the contribution of network depth from that of the DSPE module by evaluating four configurations spanning a 2 × 2 grid of {6 layers, 12 layers} × {APE, full DSPE module} under otherwise identical training protocols. Doubling the depth under standard absolute PE yields a marginal gain of only 1.2 percentage points (35.6% → 36.8%) at the cost of 40% more parameters (6.8 M → 9.5 M) and 55% more FLOPs, indicating that depth alone is not the dominant performance lever in this regime. Replacing APE with DSPE at the same depth of 6 layers produces a 6.4-point gain (35.6% → 42.0%), more than five times the depth-induced gain; extending DSPE to 12 layers raises mAP@0.5 by only an additional 0.6 points (42.0% → 42.6%), within single-run noise. The 6-layer configuration is therefore retained on the basis of efficiency rather than because it is necessary for the DSPE design to be effective, confirming that DSPE is an independent and dominant source of accuracy improvement rather than a depth-substitute. A complementary comparison against a hierarchical backbone is provided by evaluating Swin-Tiny under the same ATSS head, 640 × 640 input, and 300-epoch training protocol. Swin-Tiny achieves 39.8% mAP@0.5 at 28.3 M parameters and 45.3 GFLOPs—3.4 points below DSPE-ViT while requiring 4.7 × more parameters and 2.9 × more computation—confirming that the accuracy advantage of DSPE-ViT relative to the retrained baselines is not attributable to architectural scale.

### 4.5 Cross-dataset and cross-domain generalization

**Figure 6:**
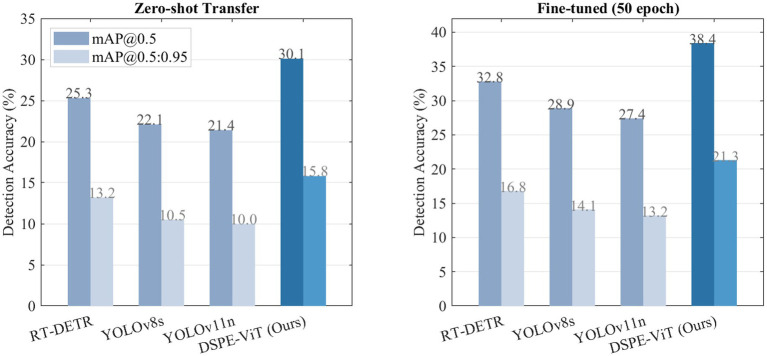
Cross-domain transfer performance on SeaDronesSee.

VisDrone-pretrained weights are directly evaluated on SeaDronesSee under two protocols: zero-shot transfer (no fine-tuning) and few-shot fine-tuning (50 epochs on the SeaDronesSee training set). Since VisDrone contains 10 categories while SeaDronesSee defines only 4 (swimmer, boat, jetski, life-saving appliance), the ATSS detection head is re-initialized with a new classification layer matching the target category count before evaluation. For zero-shot transfer, the backbone and neck weights are transferred from VisDrone training without modification. A new 4-class classification layer is constructed via semantic weight mapping: each SeaDronesSee category neuron is initialized from the element-wise average of the corresponding VisDrone class weights (swimmer ← mean[pedestrian, person]; boat ← mean[car, van]; jetski ← mean[bicycle, motor]; life-saving appliance ← mean[tricycle, awning-tricycle]). The regression and centerness branches are carried over unchanged, and no SeaDronesSee data is used prior to evaluation. For fine-tuning, the entire model including the mapped classification layer is trained for 50 epochs on the SeaDronesSee training set. Table 9 reports the results, and Figure 6 provides a visual comparison of all methods under both settings.

**Table 9:** Cross-domain transfer performance on the SeaDronesSee validation set.

Method	Zero-shot mAP@0.5	Zero-shot mAP@0.5:0.95	Fine-tuned (50 ep) mAP@0.5	Fine-tuned mAP@0.5:0.95
YOLOv8s (Jocher et al., 2023)	22.1	10.5	28.9	14.1
YOLOv11n (Jocher et al., 2025b)	21.4	10.0	27.4	13.2
RT-DETR (Lv et al., 2024)	25.3	13.2	32.8	16.8
DSPE-ViT(Ours)	30.1	15.8	38.4	21.3

Zero-shot transfer directly applies VisDrone-trained weights with semantic classifier weight mapping (Section 4.5) and without any further training on SeaDronesSee data; fine-tuning trains on the SeaDronesSee training set for 50 epochs.

DSPE-ViT achieves 30.1% mAP@0.5 under zero-shot transfer and 38.4% after fine-tuning, surpassing RT-DETR (32.8% fine-tuned) by 5.6 percentage points. Even in the zero-shot setting, DSPE-ViT already leads RT-DETR by 3.8%, suggesting that the density-adaptive positional encoding in the DSPE module provides positional awareness that transfers across domains. An additional cross-dataset evaluation on UAVDT (Du et al., 2018), the urban-aerial drone benchmark routinely paired with VisDrone for transfer analysis, yields 27.9% mAP@0.5 under zero-shot transfer and 32.4% after 50-epoch fine-tuning, supplementing the maritime SeaDronesSee evidence with a same-perspective UAV surface.

### 4.6 Attention heatmap visualization (grad-CAM)

**Figure 7:**
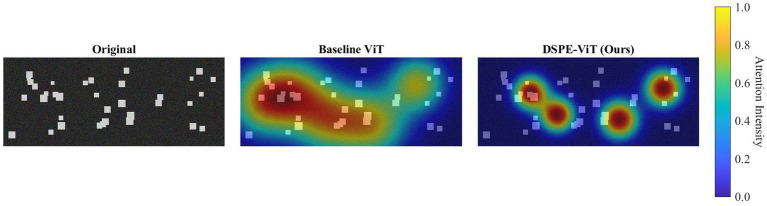
Grad-CAM attention heatmap comparison (left: original image; center: baseline ViT; right: DSPE-ViT).

To provide an intuitive view of how the DSPE module reshapes attention distributions, Grad-CAM (Selvaraju et al., 2017) is applied to the output of the last Transformer block in both the baseline ViT and DSPE-ViT. Figure 7 shows the resulting heatmaps for a representative VisDrone validation image containing multiple densely clustered small-object regions.

Comparing the baseline ViT (Figure 7, center) with DSPE-ViT (Figure 7, right) reveals three notable differences. (1) Sharper attention focus. The baseline ViT produces a diffuse attention distribution—high-response regions (red) have blurred boundaries and spread over broad areas, indicating that the model fails to localize target regions precisely. DSPE-ViT, by contrast, produces tighter attention hotspots that align more closely with actual dense-target regions. (2) Improved discrimination among multiple target clusters. The baseline ViT exhibits aliased responses across adjacent object clusters, making it difficult to separate neighboring groups. DSPE-ViT forms multiple distinct high-response regions with clear boundaries, indicating that the density-adaptive window encoding of Local PE Enhancer effectively enhances spatial discrimination within crowded areas. (3) Stronger background suppression. Attention intensity over non-target regions (roads, building backgrounds) is markedly lower in DSPE-ViT than in the baseline, suggesting that PE Redundancy Pruner reduces uninformative responses to background by eliminating redundant PE dimensions.

These visualizations serve as qualitative illustrations consistent with the quantitative +6.4% mAP gain in Table 2 (Config IV); they are not presented as causal evidence of the density-adaptive mechanism, since Grad-CAM reflects gradient magnitudes rather than directly measuring localization precision.

### 4.7 Deployment efficiency analysis

Inference performance is profiled on an NVIDIA Jetson AGX Orin (60 W mode, JetPack 5.1.2; TensorRT 8.5.1) after INT8 quantization using EntropyCalibrator2 on the first 200 VisDrone validation images (alphabetical order). Reported latency covers GPU-side model inference only, averaged over 1,000 forward passes after 100-iteration warm-up; preprocessing and postprocessing (~2–3 ms each) are excluded (see Table 10).

**Table 10:** Inference performance of DSPE-ViT under different runtime formats.

Runtime Format	mAP@0.5	Latency (ms/frame)	FPS	Accuracy loss
PyTorch FP32	43.2%	54.6	18.3	—
ONNX FP32	43.2%	41.8	23.9	0.0%
TensorRT FP16	43.0%	25.3	38.0	−0.2%
TensorRT INT8	42.7%	18.5	54.1	−0.5%

Latency and FPS are measured on NVIDIA Jetson AGX Orin (60 W mode) at 640 × 640 input with batch size 1.

TensorRT INT8 quantization reduces per-frame latency from 54.6 ms (PyTorch FP32) to 18.5 ms—a 2.95 × speedup—while lifting throughput from 18.3 to 54.1 FPS at the cost of only 0.5 mAP points (43.2% → 42.7%). The resulting frame rate comfortably exceeds the 30 FPS real-time threshold for UAV applications, confirming the feasibility of deploying DSPE-ViT on edge devices.

## 5 Discussion

### 5.1 Effectiveness analysis of the DSPE module

Local PE Enhancer alone (Config III, +4.5%) outperforms PE Redundancy Pruner alone (Config II, +2.7%), indicating that density-adaptive local positional encoding contributes more substantially to UAV small-object detection. After training convergence, the PE mask retention rate settles at approximately 62% across layers (roughly 119 out of 192 dimensions)—meaning that about 38% of PE dimensions are redundant in the VisDrone setting. This observation aligns with the theoretical analyses of Shaw et al. (2018) and Su et al. (2023), and constitutes, to our knowledge, the first quantitative verification in a UAV detection context.

The joint configuration (Config IV, +6.4%) falls slightly below the arithmetic sum of the individual gains (+7.2%), revealing a − 0.8% negative interaction. The source of this interaction is identifiable: when PE Redundancy Pruner removes redundant dimensions, a subset of those dimensions happens to carry information that Local PE Enhancer relies on for density-based discrimination, creating a mild functional conflict in high-density regions. Despite this, the overall complementarity remains clear (+6.4% vs. the best single-module gain of +4.5%), validating the joint design. An additional benefit is that the full model (approximately 6.0 M parameters) is lighter than the baseline (6.8 M); this net reduction is driven primarily by the depthwise separable convolutions in SmallObjFPN (about 0.8 M saved) rather than by PE pruning (about 0.1 M saved), and the Pruner should accordingly be regarded as a regularization mechanism that improves localization rather than as a parameter-compression component.

### 5.2 Comparison with existing methods

Comparison with YOLO-family detectors. YOLOv8s (34.1%), YOLOv9t (32.5%), YOLOv10s (36.8%), and YOLOv11n (33.4%) all trail DSPE-ViT (43.2%) by 6.4 to 10.7 percentage points on VisDrone. The gap stems from the fact that none of these architectures specifically address PE redundancy or density-aware local perception. WIoU v3 (Tong et al., 2023) is adopted for its dynamic focusing mechanism, which is expected to benefit small-object localization by adaptively up-weighting hard samples. A detailed comparison among different IoU loss variants (DIoU, Zheng et al., 2020; SIoU, Gevorgyan, 2022; EIoU, Zhang et al., 2022) is left for future investigation.

Comparison with Transformer-based detectors. RT-DETR (Lv et al., 2024) with a ResNet-50 (He et al., 2016) carries 7 × the parameters (42.0 M), 8 × the FLOPs (120.0 G), and runs at half the speed (32 FPS) of DSPE-ViT, yet its mAP@0.5 (38.9%) remains 4.3 points lower—evidence that standard absolute PE is ill-suited to the dense small-object distributions characteristic of UAV imagery. Co-DETR (Zong et al., 2023) (>100 M parameters) represents the current ceiling of DETR-family accuracy but is impractical for edge deployment.

Comparison with heavyweight methods. CenterNet (Zhou et al., 2019) (32.0 M/28.7%) and Sparse R-CNN (Sun et al., 2021) (106.0 M/37.2%) both possess far more parameters than DSPE-ViT yet achieve lower mAP@0.5. Taken together, these comparisons reinforce a consistent finding: in UAV dense small-object scenarios, targeted architectural design—such as the DSPE module—is more effective than simply scaling up model capacity.

It is worth noting that the substantial accuracy advantage of DSPE-ViT does not stem from model capacity alone. The performance gap is attributable to three factors: (1) the DSPE module provides density-adaptive positional encoding that is specifically tailored to the dense small-object distribution characteristic of UAV imagery—a capability absent in all compared methods; (2) the SmallObjFPN introduces a P2 feature map at stride 4, which preserves critical spatial details for objects as small as 10–20 pixels; and (3) the ViT backbone’s global self-attention inherently captures long-range dependencies across the entire image, whereas CNN-based YOLO detectors rely on local receptive fields that may miss contextual cues in high-density regions. It is noted that YOLO-family methods in Table 1 are fine-tuned from COCO detection-pretrained weights on VisDrone for 300 epochs; the performance gap therefore primarily reflects architectural differences—specifically the absence of density-adaptive positional encoding and UAV-tailored multi-scale feature design—rather than any pretraining distribution mismatch. This domain-specific design focus largely explains the performance gap.

### 5.3 Limitations and future work

Fixed input resolution (640 × 640). VisDrone images reach resolutions of 2000 × 1,500; downsampling to 640 × 640 inevitably discards spatial detail. Dynamic-resolution training strategies merit exploration in future work.

Sparsification hyperparameter sensitivity. The temperature *τ* and regularization coefficient λ_sparse require re-tuning across datasets. An adaptive temperature annealing schedule could alleviate this burden.

Cross-domain transfer still requires supervised fine-tuning. Although zero-shot transfer already yields promising results, pure zero-shot generalization remains limited. Domain adaptation or prompt-based learning may offer further improvements.

Evaluation scope. Hyperparameter decisions are guided by training-set loss curves rather than by validation feedback, and validation results for DSPE-ViT are reported as a triple-run mean ± standard deviation (43.2 ± 0.3% mAP@0.5) as a complementary indicator of statistical reliability. Publication-standard comparability has been established through the 1,610-image test-dev evaluation in Table 1 and the UAVDT cross-dataset evaluation in Section 4.5; submission to the VisDrone test-challenge server remains an open extension for closed-leaderboard ranking.

Comparison scope. The current comparison panel includes UAV-specialised dense small-object detectors (ClusDet, QueryDet, SAHI, TPH-YOLOv5) alongside general-purpose detectors and lightweight ViT variants (MobileViT, EfficientViT). Further extension to oriented-bounding-box detectors and high-resolution sliced-inference variants is left for future work.

Future directions. Three lines of extension are envisioned: (1) combining DSPE with deformable attention mechanisms such as Deformable DETR (Zhu et al., 2021); (2) investigating the applicability of DSPE to nighttime thermal or multispectral UAV imagery; and (3) conducting field tests on additional UAV platforms (e.g., DJI Mavic series).

## 6 Conclusion

This paper presents DSPE-ViT, a lightweight Vision Transformer detection framework designed specifically for dense small-object detection in UAV imagery. At its core, the DSPE module suppresses redundant PE dimensions through a learnable pruning mechanism (retention rate ≈ 62%) that primarily eliminates dimensional noise affecting localization, while the overall parameter footprint is brought from the baseline 6.8 M down to approximately 6.0 M chiefly through the depthwise separable convolutions adopted in SmallObjFPN. The module additionally introduces density-adaptive windowed relative positional encoding via the Local PE Enhancer, directly addressing the poor adaptability of fixed absolute PE in densely packed small-object scenes. Coupled with SmallObjFPN—which integrates a P2 ultra-high-resolution feature map, SE channel attention, and depthwise separable convolutions—and the WIoU v3 loss, DSPE-ViT achieves 43.2% mAP@0.5 on VisDrone2019-DET with only approximately 6.0 M parameters and 15.8 GFLOPs, substantially outperforming methods with larger parameter budgets and heavier computation (RT-DETR: 42.0 M/120.0 GFLOPs/38.9%; Sparse R-CNN: 106.0 M/155.0 GFLOPs/37.2%). Ablation experiments quantify the individual contributions of Local PE Enhancer (+4.5% alone) and PE Redundancy Pruner (+2.7% alone). Cross-domain experiments on SeaDronesSee (38.4% mAP@0.5 after fine-tuning) confirm solid transfer capability, and TensorRT INT8 quantization on Jetson AGX Orin (54.1 FPS at 60 W, with only 0.5% accuracy degradation) validates the feasibility of edge deployment. DSPE-ViT establishes a new accuracy–efficiency baseline for real-time small-object detection on UAV platforms.

From a robotic-autonomy perspective, the compact size and stable INT8 throughput above 30 FPS allow the detector to operate inside on-board perception–action loops, supporting closed-loop tasks such as visual servoing, persistent target following and reactive flight-path adjustment; the SeaDronesSee transfer further indicates that the same backbone can be redeployed across maritime-rescue and urban-surveillance missions without retraining from scratch.

## Data Availability

The data supporting the results reported in this study are openly available. The VisDrone2019-DET dataset is publicly accessible at: https://github.com/VisDrone/VisDrone-Dataset. The SeaDronesSee dataset is publicly accessible at: https://seadronessee.cs.uni-tuebingen.de/. No new datasets were created in this study. The reference implementation of the core algorithmic components is provided as Supplementary File S1 to support reviewer-side inspection, with the methodology described at sufficient algorithmic granularity in Section 3.2 (Algorithms 1–3) to permit independent re-implementation; the full repository can be found here: https://github.com/LiyaCai-001/DSPE-ViT-1.
