# Supplementary material for: DSPE-ViT: a lightweight vision transformer with dynamic sparse positional encoding for dense small object detection in UAV imagery
Source: Front Neurorobot. 2026 Jun 16;20:1849093. doi: 10.3389/fnbot.2026.1849093 (PMC13316741; doi:10.3389/fnbot.2026.1849093)
Supplement: Supplementary file 1 [file Supplementary_file_1.docx]

Supplementary File S1 — Reference Implementations

Concatenated source listings of the three core algorithmic components of DSPE-ViT, provided to peer reviewers as self-contained reference implementations. The files are MMDetection 3.x compatible and can be dropped into a custom mmdet_custom/ package without modification. Each file is presented below under a heading that gives its original path within the supplementary package.

**Module mapping:** DSPE module (Section 3.2, Algorithms 1–3) → dspe_module.py + redundancy_pruner.py + local_pe_enhancer.py; SmallObjFPN neck (Section 3.3) → small_obj_fpn.py; WIoU-v3 loss (Section 3.4) → wiou_loss.py.

# dspe_module/dspe_module.py

# Copyright (c) 2024. DSPE-ViT Authors.
# ============================================================
# dspe_module.py
# Dynamic Sparse Positional Encoding (DSPE) Integration Module
# Fuses redundancy pruning + local enhancement into a unified plug-and-play PE module
# Corresponds to paper Section 3.2 "Overall Optimized Positional Encoding Scheme"
# ============================================================

import torch
import torch.nn as nn
import torch.nn.functional as F
from mmengine.model import BaseModule
from mmdet.registry import MODELS

from .redundancy_pruner import PERedundancyPruner
from .local_pe_enhancer import LocalPEEnhancer


@MODELS.register_module()
class DSPEModule(BaseModule):
 """Dynamic Sparse Positional Encoding (DSPE) Module.

 The core innovation module proposed in this paper, unifying two design
 strategies into a plug-and-play component:
 1. PERedundancyPruner: soft-mask pruning of redundant PE dimensions
 2. LocalPEEnhancer: density-adaptive local relative position enhancement

 Comparison with standard 2D learnable PE:
 +------------------+----------+------------------+---------------+
 | Module | Params | Small Obj Acc | Spatial Aware |
 +------------------+----------+------------------+---------------+
 | Learned 2D PE | N x D | Moderate | Fixed |
 | RoPE | 0 | Good | Relative |
 | DSPE (Ours) | ~(2W-1)^2 x H + D/8 | Best | Dynamic+Local |
 +------------------+----------+------------------+---------------+

 Args:
 embed_dim (int): Embedding dimension. Default 384.
 num_heads (int): Number of attention heads. Default 6.
 img_size (int): Input image size. Default 640.
 patch_size (int): Patch size. Default 16.
 keep_ratio (float): PE dimension retention ratio. Default 0.5.
 window_size (int): Local window size in patches. Default 4.
 density_lambda (float): Density scaling coefficient. Default 0.5.
 sparsity_weight (float): Sparsity loss weight. Default 0.01.
 use_density_scale (bool): Whether to enable density scaling. Default True.
 """

 def __init__(
 self,
 embed_dim: int = 192, # Corresponds to ViT-Tiny backbone
 num_heads: int = 3,
 img_size: int = 640,
 patch_size: int = 16,
 keep_ratio: float = 0.5,
 window_size: int = 4,
 density_lambda: float = 0.5,
 sparsity_weight: float = 0.01,
 use_density_scale: bool = True,
 init_cfg=None,
 ):
 super().__init__(init_cfg=init_cfg)
 self.embed_dim = embed_dim
 self.sparsity_weight = sparsity_weight

 # Patch grid dimensions
 self.H = img_size // patch_size # e.g., 640//16 = 40
 self.W = img_size // patch_size
 self.num_patches = self.H * self.W # e.g., 1600

 # ── Standard global learnable PE (base, then stacked with DSPE) ──────────
 self.base_pe = nn.Parameter(
 torch.zeros(1, self.num_patches, embed_dim)
 )
 nn.init.trunc_normal_(self.base_pe, std=0.02)

 # ── Redundancy pruning module ─────────────────────────────────────────────
 self.pruner = PERedundancyPruner(
 embed_dim=embed_dim,
 keep_ratio=keep_ratio,
 )

 # ── Local enhancement module ──────────────────────────────────────────────
 self.local_enhancer = LocalPEEnhancer(
 embed_dim=embed_dim,
 num_heads=num_heads,
 window_size=window_size,
 density_lambda=density_lambda,
 use_density_scale=use_density_scale,
 )

 # ── Fusion weights (learnable, balance pruned PE and local enhancement) ───
 self.fusion_weight = nn.Parameter(torch.tensor([0.8, 0.2]))

 def forward(
 self,
 tokens: torch.Tensor,
 H: int = None,
 W: int = None,
 ) -> tuple:
 """Forward pass.

 Args:
 tokens (Tensor): Patch token sequence, shape [B, N, D].
 H (int): Patch grid height (optional, defaults to value at init).
 W (int): Patch grid width (optional, defaults to value at init).

 Returns:
 tuple:
 - tokens_with_pe (Tensor): Tokens with DSPE applied, shape [B, N, D].
 - loss_dict (dict): Contains sparsity loss (used during training).
 """
 B, N, D = tokens.shape
 H = H if H is not None else self.H
 W = W if W is not None else self.W

 # base_pe shape is [1, N, D]; pass directly to pruner (pruner uses only pe[0] for importance)
 pe_pruned, gate = self.pruner(self.base_pe) # [1, N, D], [D]
 pe_pruned = pe_pruned.expand(B, -1, -1) # [B, N, D]

 local_pe = self.local_enhancer(tokens, H, W) # [B, N, D]

 fusion_w = F.softmax(self.fusion_weight, dim=0)
 tokens_with_pe = tokens + fusion_w[0] * pe_pruned + fusion_w[1] * local_pe

 if self.training:
 return tokens_with_pe, {
 'loss_pe_sparsity': self.sparsity_weight * self.pruner.get_sparsity_loss(gate)
 }
 return tokens_with_pe, {}

 def flops(self) -> int:
 """Estimate floating-point operations (FLOPs) for the DSPE module.

 Returns:
 int: Approximate FLOPs count.
 """
 N = self.num_patches
 D = self.embed_dim
 W2 = self.local_enhancer.window_size ** 2

 # Pruner: importance proj D->D/8 + D/8->D (two linear layers)
 pruner_flops = N * (D * (D // 8) + (D // 8) * D)

 # Local enhancer: relative bias lookup + proj per window token
 enhancer_flops = N * W2 * self.local_enhancer.num_heads

 # Fusion: element-wise weighted sum
 fusion_flops = N * D * 2

 return pruner_flops + enhancer_flops + fusion_flops

 def extra_repr(self) -> str:
 return (
 f'embed_dim={self.embed_dim}, '
 f'num_patches={self.num_patches}, '
 f'keep_ratio={self.pruner.keep_ratio}, '
 f'window_size={self.local_enhancer.window_size}'
 )

# dspe_module/redundancy_pruner.py

# Copyright (c) 2024. DSPE-ViT Authors.
# ============================================================
# redundancy_pruner.py
# Redundant Positional Encoding Dimension Pruner
# Core Innovation 1: Automatically identify and soft-mask low-utility PE
# dimensions via learnable importance scoring.
# Corresponds to paper Section 3.2 "Pruning Strategy for Redundant PE Dimensions"
# ============================================================

import torch
import torch.nn as nn
import torch.nn.functional as F
from mmengine.model import BaseModule
from mmdet.registry import MODELS


@MODELS.register_module()
class PERedundancyPruner(BaseModule):
 """Positional Encoding Redundancy Pruner.

 Scores the importance of each PE dimension and applies a learnable
 soft-gating mask to suppress low-information redundant dimensions,
 achieving lightweight compression of PE.

 Core idea:
 1. Use channel attention to assign importance scores to each PE dimension
 2. Apply sigmoid soft-gating for differentiable dimension selection
 3. Automatically learn which dimensions contribute to small-object localization
 4. At inference, hard thresholding can be enabled for true sparsity

 Args:
 embed_dim (int): Embedding dimension D of the positional encoding. Default 384.
 keep_ratio (float): Target retention ratio, controls number of active dims. Default 0.5.
 temperature (float): Sigmoid temperature, controls gate hardness. Default 1.0.
 init_bias (float): Gate bias initialization; positive values bias towards retaining
 more dimensions. Default 0.5.

 Math:
 Importance score: s = MeanPool(|PE|) in R^D
 Gate weight: g = sigmoid((W_proj(s) + b) / tau) in [0,1]^D
 Pruned output: PE_pruned = PE * g

 References:
 - Network Slimming (Liu et al., 2017)
 - Soft Filter Pruning (He et al., 2018)
 """

 def __init__(
 self,
 embed_dim: int = 192, # Corresponds to ViT-Tiny
 keep_ratio: float = 0.5,
 temperature: float = 1.0,
 init_bias: float = 0.5,
 init_cfg=None,
 ):
 super().__init__(init_cfg=init_cfg)
 self.embed_dim = embed_dim
 self.keep_ratio = keep_ratio
 self.temperature = temperature

 # Importance scoring projection: D -> D
 # Bottleneck structure to reduce parameter count
 bottleneck = max(embed_dim // 8, 16)
 self.importance_proj = nn.Sequential(
 nn.Linear(embed_dim, bottleneck, bias=False),
 nn.LayerNorm(bottleneck),
 nn.GELU(),
 nn.Linear(bottleneck, embed_dim, bias=True),
 )

 # Initialize: bias towards retaining more dimensions (positive bias)
 nn.init.constant_(self.importance_proj[-1].bias, init_bias)
 nn.init.xavier_uniform_(self.importance_proj[-1].weight, gain=0.02)

 self.register_buffer('sparsity_ema', torch.zeros(1))

 _EMA_MOMENTUM = 0.99

 def forward(self, pe: torch.Tensor) -> tuple:
 """Forward pass.

 Args:
 pe (Tensor): Positional encoding tensor, shape [B, N, D] or [1, N, D].
 When called from DSPEModule, input is [1, N, D] (base_pe not expanded),
 avoiding redundant mean computation across repeated batch dims.

 Returns:
 tuple:
 - pe_pruned (Tensor): Pruned PE, same shape as input.
 - gate (Tensor): Gate weights, shape [D], for analysis and visualization.
 """
 # Compute importance score from first sample only: base_pe is identical across batch
 importance = pe[0].abs().mean(dim=0) # [D]

 gate_logit = self.importance_proj(importance.unsqueeze(0)).squeeze(0) # [D]
 gate = torch.sigmoid(gate_logit / self.temperature) # [D]

 # Hard thresholding (can be enabled at inference; uses soft gate during training)
 # if not self.training:
 # threshold = gate.kthvalue(int(self.embed_dim * (1 - self.keep_ratio))).values
 # gate = (gate >= threshold).float()

 pe_pruned = pe * gate[None, None, :] # [B, N, D]

 if self.training:
 current_sparsity = (gate < 0.5).float().mean()
 self.sparsity_ema.mul_(self._EMA_MOMENTUM).add_(
 current_sparsity.detach(), alpha=(1 - self._EMA_MOMENTUM)
 )

 return pe_pruned, gate

 def get_sparsity_loss(self, gate: torch.Tensor) -> torch.Tensor:
 """Compute sparsity regularization loss to encourage the gate towards the target sparsity.

 Args:
 gate (Tensor): Gate weights [D].

 Returns:
 Tensor: Sparsity loss (scalar).
 """
 return F.l1_loss(gate.mean(), gate.new_tensor(self.keep_ratio))

 def get_active_dims(self) -> int:
 """Return the approximate number of currently active (important) dimensions."""
 return int(self.embed_dim * self.keep_ratio)

 def extra_repr(self) -> str:
 return (
 f'embed_dim={self.embed_dim}, '
 f'keep_ratio={self.keep_ratio}, '
 f'temperature={self.temperature}'
 )

# dspe_module/local_pe_enhancer.py

# Copyright (c) 2024. DSPE-ViT Authors.
# ============================================================
# local_pe_enhancer.py
# Local Positional Encoding Enhancer for Small Object Dense Regions
# Core Innovation 2: Inject stronger spatial priors into small-object-dense
# regions to improve localization accuracy.
# Corresponds to paper Section 3.2 "Local Relative Position Enhancement
# for Small Object Dense Regions"
# ============================================================

import torch
import torch.nn as nn
import torch.nn.functional as F
from mmengine.model import BaseModule
from mmdet.registry import MODELS


@MODELS.register_module()
class LocalPEEnhancer(BaseModule):
 """Local Positional Encoding Enhancer for Small Object Dense Regions.

 Combines global absolute PE with intra-window relative position bias,
 and dynamically scales local PE strength according to regional small-object
 density, enabling finer spatial perception in dense small-object regions.

 Design highlights:
 1. Window partitioning: divide patch tokens into W×W local windows
 2. Relative position bias: build (2W-1)×(2W-1) bias table per window
 3. Density-adaptive scaling: estimate target density from token
 activation norm within each window
 4. Lightweight: relative bias table has only (2W-1)² parameters,
 far fewer than global PE

 Args:
 embed_dim (int): Embedding dimension. Default 384.
 num_heads (int): Number of attention heads (bias is per-head). Default 6.
 window_size (int): Local window size in patches. Default 4.
 density_lambda (float): Density scaling coefficient lambda. Default 0.5.
 use_density_scale (bool): Whether to enable density-adaptive scaling. Default True.

 Math:
 Relative position index in window:
 Delta_i = i_row - j_row + (W-1), Delta_j = i_col - j_col + (W-1)
 Bias lookup:
 bias[q,k] = B[Delta_i*(2W-1) + Delta_j] in R^{heads}
 Density estimation:
 density_w = mean(||token_w||_2) / global_mean
 Enhanced output:
 local_pe_w = (1 + lambda * density_w) * relative_bias_w
 Final PE:
 PE_enhanced = PE_pruned + local_pe_w (after projection)

 References:
 - Swin Transformer (Liu et al., 2021): window-based relative PE
 - Twins-SVT (Chu et al., 2021): local-global PE
 """

 def __init__(
 self,
 embed_dim: int = 192, # Corresponds to ViT-Tiny
 num_heads: int = 3,
 window_size: int = 4,
 density_lambda: float = 0.5,
 use_density_scale: bool = True,
 init_cfg=None,
 ):
 super().__init__(init_cfg=init_cfg)
 self.embed_dim = embed_dim
 self.num_heads = num_heads
 self.window_size = window_size
 self.density_lambda = density_lambda
 self.use_density_scale = use_density_scale

 # ── Relative position bias table ───────────────────────────────────────
 # Size: (2*window_size-1) x (2*window_size-1) x num_heads
 self.relative_position_bias_table = nn.Parameter(
 torch.zeros(
 (2 * window_size - 1) ** 2,
 num_heads
 )
 )
 nn.init.trunc_normal_(self.relative_position_bias_table, std=0.02)

 # Pre-compute relative position index (no gradient)
 self._build_relative_position_index(window_size)

 # ── Density-aware projection ────────────────────────────────────────────
 # Project head-wise bias to embed_dim space so it can be added to PE
 self.bias_proj = nn.Linear(num_heads, embed_dim, bias=False)
 nn.init.trunc_normal_(self.bias_proj.weight, std=0.02)

 # Gate controlling overall local enhancement strength
 # Initialized to 0 -> gradual enhancement, avoids instability early in training
 self.local_gate = nn.Parameter(torch.zeros(1))
 # Cache static bias projection when bias_proj parameters are unchanged
 self._cached_token_bias_emb = None

 def _build_relative_position_index(self, window_size: int):
 """Pre-compute relative position indices for all token pairs in a window."""
 W = window_size
 # Generate coordinate grid
 coords_h = torch.arange(W)
 coords_w = torch.arange(W)
 coords = torch.stack(torch.meshgrid(coords_h, coords_w, indexing='ij')) # [2, W, W]
 coords_flat = coords.flatten(1) # [2, W²]

 # Compute relative positions for all token pairs
 relative_coords = coords_flat[:, :, None] - coords_flat[:, None, :] # [2, W², W²]
 relative_coords = relative_coords.permute(1, 2, 0) # [W², W², 2]

 # Normalize to non-negative integer indices
 relative_coords[:, :, 0] += (W - 1)
 relative_coords[:, :, 1] += (W - 1)
 relative_coords[:, :, 0] *= (2 * W - 1)
 relative_position_index = relative_coords.sum(-1) # [W², W²]

 self.register_buffer(
 'relative_position_index',
 relative_position_index
 )

 def _estimate_density(self, tokens: torch.Tensor, H: int, W: int) -> torch.Tensor:
 """Estimate small-object density per window (based on normalized L2 norm of token activations)."""
 B, N, D = tokens.shape
 Wh = self.window_size
 num_win_h, num_win_w = H // Wh, W // Wh
 feat_win = (
 tokens.reshape(B, H, W, D)
 .reshape(B, num_win_h, Wh, num_win_w, Wh, D)
 .permute(0, 1, 3, 2, 4, 5)
 ) # [B, nH, nW, Wh, Wh, D]
 win_activation = feat_win.norm(dim=-1).mean(dim=(-2, -1)) # [B, nH, nW]
 global_mean = win_activation.mean(dim=(1, 2), keepdim=True) + 1e-6
 return win_activation / global_mean

 def _get_token_bias_emb(self) -> torch.Tensor:
 """Return the static position bias projection per token in a window (cached to avoid recomputation).

 rel_bias / self_bias / context_bias depend only on fixed parameters, not
 on input tokens, so results are constant when parameters are unchanged,
 and can be safely cached. During training, gradients back-propagate
 through bias_proj.weight and relative_position_bias_table normally;
 the cache is invalidated after each optimizer step.
 """
 if self._cached_token_bias_emb is None or self.training:
 W2 = self.window_size ** 2
 rel_bias = self.relative_position_bias_table[
 self.relative_position_index.view(-1)
 ].view(W2, W2, -1) # [W², W², heads]
 # diagonal: self-position bias at i==j -> [heads, W²] -> [W², heads]
 self_bias = rel_bias.diagonal(dim1=0, dim2=1).permute(1, 0)
 context_bias = rel_bias.mean(dim=1) # [W², heads]
 token_bias = (self_bias + context_bias) / 2.0 # [W², heads]
 result = self.bias_proj(token_bias) # [W², D]
 if not self.training:
 self._cached_token_bias_emb = result.detach()
 else:
 return result
 return self._cached_token_bias_emb

 def forward(
 self,
 tokens: torch.Tensor,
 H: int,
 W: int,
 ) -> torch.Tensor:
 """Forward pass.

 Args:
 tokens (Tensor): Patch token sequence, shape [B, N, D], N = H * W.
 H (int): Feature map height in patches.
 W (int): Feature map width in patches.

 Returns:
 Tensor: Local position enhancement bias, shape [B, N, D], to be added to PE.
 """
 B, N, D = tokens.shape
 Wh = self.window_size
 assert H % Wh == 0 and W % Wh == 0, (
 f'H={H} and W={W} must be divisible by window_size={Wh}'
 )
 num_win_h = H // Wh
 num_win_w = W // Wh

 # token_bias_emb: [W², D] = [Wh*Wh, D]
 token_bias_emb = self._get_token_bias_emb()

 if self.use_density_scale:
 density = self._estimate_density(tokens, H, W) # [B, nH, nW]
 scale = 1.0 + self.density_lambda * density # [B, nH, nW]
 # Avoid materialising [B, nH, nW, W², D] by broadcasting directly
 # into [B, nH, nW, Wh, Wh, D] without the tile step
 bias_scaled = (
 scale.view(B, num_win_h, num_win_w, 1, 1, 1)
 * token_bias_emb.view(1, 1, 1, Wh, Wh, D)
 ) # [B, nH, nW, Wh, Wh, D]
 else:
 bias_scaled = token_bias_emb.view(1, 1, 1, Wh, Wh, D).expand(
 B, num_win_h, num_win_w, -1, -1, -1
 ) # [B, nH, nW, Wh, Wh, D]

 local_pe = (
 bias_scaled
 .permute(0, 1, 3, 2, 4, 5) # [B, nH, Wh, nW, Wh, D]
 .reshape(B, H * W, D)
 ) # [B, N, D]

 return torch.tanh(self.local_gate) * local_pe

 def extra_repr(self) -> str:
 return (
 f'embed_dim={self.embed_dim}, '
 f'num_heads={self.num_heads}, '
 f'window_size={self.window_size}, '
 f'density_lambda={self.density_lambda}'
 )

# small_obj_fpn/small_obj_fpn.py

# Copyright (c) 2024. DSPE-ViT Authors.
# ============================================================
# small_obj_fpn.py
# Small Object Enhanced FPN Neck
# Builds on standard FPN with the following additions:
# 1. P2 ultra-high-resolution output layer (stride=4, dedicated to <20px small objects)
# 2. Per-level channel attention (SE block) to enhance small-object feature responses
# 3. Depthwise separable convolutions replacing standard 3x3 Conv (fewer parameters)
# Corresponds to paper Section 3.3 "Overall Improved Model Architecture"
# ============================================================

import torch
import torch.nn as nn
import torch.nn.functional as F
from mmengine.model import BaseModule
from mmdet.registry import MODELS


class DepthwiseSepConv(nn.Module):
 """Depthwise Separable Convolution = Depthwise Conv + Pointwise Conv (lightweight)."""

 def __init__(self, in_ch, out_ch, kernel_size=3, stride=1, padding=1):
 super().__init__()
 self.dw = nn.Conv2d(in_ch, in_ch, kernel_size, stride, padding, groups=in_ch, bias=False)
 self.pw = nn.Conv2d(in_ch, out_ch, 1, bias=False)
 self.bn = nn.BatchNorm2d(out_ch)
 self.act = nn.GELU()

 def forward(self, x):
 return self.act(self.bn(self.pw(self.dw(x))))


class SEBlock(nn.Module):
 """Squeeze-and-Excitation channel attention (enhances small-object feature responses)."""

 def __init__(self, channels, reduction=8):
 super().__init__()
 mid = max(channels // reduction, 8)
 self.fc = nn.Sequential(
 nn.AdaptiveAvgPool2d(1),
 nn.Flatten(),
 nn.Linear(channels, mid, bias=False),
 nn.GELU(),
 nn.Linear(mid, channels, bias=False),
 nn.Sigmoid(),
 )

 def forward(self, x):
 w = self.fc(x).view(x.shape[0], -1, 1, 1)
 return x * w


@MODELS.register_module()
class SmallObjFPN(BaseModule):
 """Small Object Enhanced FPN Neck.

 Two improvements over standard FPN:
 - P2 layer: stride=4 ultra-high-resolution feature map for <20px small objects
 - SE Channel Attention: per-level enhancement of small-object-relevant channels
 - Depthwise separable convolutions: lightweight fusion of lateral + top-down features

 Input (4-scale multi-scale output from DSPEViT):
 in_channels = [embed_dim] * 4, shapes from large to small
 Typical: [(B,384,160,160), (B,384,80,80), (B,384,40,40), (B,384,20,20)]
 for patch_size=4; for patch_size=16: 40, 20, 10, 5

 Output (P2-P5, 4 scales):
 [(B, out_channels, H/4, W/4),
 (B, out_channels, H/8, W/8),
 (B, out_channels, H/16, W/16),
 (B, out_channels, H/32, W/32)]

 Args:
 in_channels (list[int]): Channel count per input feature map. Default [384,384,384,384].
 out_channels (int): FPN output channels. Default 256.
 num_outs (int): Number of output feature maps (including extra P5+). Default 5.
 use_se (bool): Whether to use SE channel attention. Default True.
 se_reduction (int): SE channel reduction ratio. Default 8.
 """

 def __init__(
 self,
 in_channels=(384, 384, 384, 384),
 out_channels: int = 256,
 num_outs: int = 5,
 use_se: bool = True,
 se_reduction: int = 8,
 init_cfg=None,
 ):
 super().__init__(init_cfg=init_cfg)
 self.in_channels = in_channels
 self.out_channels = out_channels
 self.num_outs = num_outs
 self.use_se = use_se
 num_ins = len(in_channels)

 # ── Lateral 1x1 Conv (channel alignment) ─────────────────────────────────
 self.lateral_convs = nn.ModuleList([
 nn.Sequential(
 nn.Conv2d(in_ch, out_channels, 1, bias=False),
 nn.BatchNorm2d(out_channels),
 )
 for in_ch in in_channels
 ])

 # ── FPN output layers (depthwise separable) ───────────────────────────────
 self.fpn_convs = nn.ModuleList([
 DepthwiseSepConv(out_channels, out_channels, 3, 1, 1)
 for _ in range(num_ins)
 ])

 # ── SE channel attention (optional) ───────────────────────────────────────
 if use_se:
 self.se_blocks = nn.ModuleList([
 SEBlock(out_channels, reduction=se_reduction)
 for _ in range(num_ins)
 ])

 # ── Extra downsampling layers (P6/P7 for RetinaNet / FCOS) ───────────────
 extra_convs = []
 for _ in range(num_outs - num_ins):
 extra_convs.append(DepthwiseSepConv(out_channels, out_channels, 3, 2, 1))
 self.extra_convs = nn.ModuleList(extra_convs)

 def forward(self, inputs: tuple) -> tuple:
 """Forward pass.

 Args:
 inputs (tuple[Tensor]): Multi-scale feature maps from backbone, shallow to deep.

 Returns:
 tuple[Tensor]: FPN output feature maps (P2, P3, P4, P5[, P6, ...]).
 """
 assert len(inputs) == len(self.in_channels)

 # ── Step 1: Lateral projection ────────────────────────────────────────────
 laterals = [conv(x) for conv, x in zip(self.lateral_convs, inputs)]

 # ── Step 2: Top-down fusion (deep to shallow) ─────────────────────────────
 for i in range(len(laterals) - 1, 0, -1):
 upsampled = F.interpolate(
 laterals[i], size=laterals[i - 1].shape[2:], mode='nearest'
 )
 laterals[i - 1] = laterals[i - 1] + upsampled

 # ── Step 3: FPN output convolutions + SE enhancement ─────────────────────
 outs = []
 for i, (fpn_conv, feat) in enumerate(zip(self.fpn_convs, laterals)):
 feat = fpn_conv(feat)
 if self.use_se:
 feat = self.se_blocks[i](feat)
 outs.append(feat)

 # ── Step 4: Extra downsampling layers ─────────────────────────────────────
 x = outs[-1]
 for extra_conv in self.extra_convs:
 x = extra_conv(x)
 outs.append(x)

 return tuple(outs)

# wiou_v3_loss/wiou_loss.py

# Copyright (c) 2024. DSPE-ViT Authors.
# ============================================================
# wiou_loss.py
# WIoU v3 Loss Function Implementation
# Corresponds to paper Section 3.4 "Loss Function Design — IoU Variant for Small Objects"
# Reference: Wise-IoU: Bounding Box Regression Loss with Dynamic Focusing Mechanism
# (Tong et al., arXiv 2301.10051, 2023)
# ============================================================

import torch
import torch.nn as nn
from mmdet.registry import MODELS
from mmdet.models.losses.utils import weighted_loss


def bbox_overlaps_wiou(pred: torch.Tensor, target: torch.Tensor,
 eps: float = 1e-7) -> torch.Tensor:
 """Compute WIoU v3 loss between predicted and target boxes (element-wise).

 Key contributions of WIoU:
 1. Dynamic focusing coefficient beta:
 Computed from geometric factor r (distance ratio of anchor to target box),
 focusing gradients onto hard samples.
 2. Smart gradient allocation: beta = exp(rho^2 / (rho_hat^2 + eps))
 - When pred is close to target: beta -> 1 (normal gradient)
 - When pred deviates from target: beta > 1 (amplified gradient, focuses on hard samples)
 3. More small-object friendly than CIoU/DIoU:
 Numerator contains only center-point distance; no aspect-ratio penalty
 (better when small-object aspect ratios are unstable)

 Args:
 pred (Tensor): Predicted boxes [N, 4], format (x1, y1, x2, y2).
 target (Tensor): Target boxes [N, 4], format (x1, y1, x2, y2).
 eps (float): Numerical stability constant.

 Returns:
 Tensor: WIoU loss values [N], range [0, 1] (lower is better).
 """
 # ── Predicted / target box basic quantities ───────────────────────────────
 pred_x1, pred_y1, pred_x2, pred_y2 = pred.unbind(-1)
 tgt_x1, tgt_y1, tgt_x2, tgt_y2 = target.unbind(-1)

 pred_w = (pred_x2 - pred_x1).clamp(min=0)
 pred_h = (pred_y2 - pred_y1).clamp(min=0)
 tgt_w = (tgt_x2 - tgt_x1).clamp(min=0)
 tgt_h = (tgt_y2 - tgt_y1).clamp(min=0)

 pred_area = pred_w * pred_h
 tgt_area = tgt_w * tgt_h

 # ── Intersection ──────────────────────────────────────────────────────────
 inter_x1 = torch.max(pred_x1, tgt_x1)
 inter_y1 = torch.max(pred_y1, tgt_y1)
 inter_x2 = torch.min(pred_x2, tgt_x2)
 inter_y2 = torch.min(pred_y2, tgt_y2)

 inter_w = (inter_x2 - inter_x1).clamp(min=0)
 inter_h = (inter_y2 - inter_y1).clamp(min=0)
 inter_area = inter_w * inter_h

 # ── IoU ───────────────────────────────────────────────────────────────────
 union = pred_area + tgt_area - inter_area + eps
 iou = inter_area / union # [N]

 # ── Minimum enclosing box (for normalized distance) ───────────────────────
 enclose_x1 = torch.min(pred_x1, tgt_x1)
 enclose_y1 = torch.min(pred_y1, tgt_y1)
 enclose_x2 = torch.max(pred_x2, tgt_x2)
 enclose_y2 = torch.max(pred_y2, tgt_y2)
 enclose_w = (enclose_x2 - enclose_x1).clamp(min=eps)
 enclose_h = (enclose_y2 - enclose_y1).clamp(min=eps)
 c2 = enclose_w ** 2 + enclose_h ** 2 # Squared diagonal of enclosing box

 # ── Squared center-point distance ─────────────────────────────────────────
 pred_cx = (pred_x1 + pred_x2) / 2
 pred_cy = (pred_y1 + pred_y2) / 2
 tgt_cx = (tgt_x1 + tgt_x2) / 2
 tgt_cy = (tgt_y1 + tgt_y2) / 2
 rho2 = (pred_cx - tgt_cx) ** 2 + (pred_cy - tgt_cy) ** 2 # [N]

 # ── WIoU v3 dynamic focusing coefficient beta ─────────────────────────────
 # rho_hat^2: normalized distance from target box center to enclosing box center
 # (proxy for anchor quality); target-to-enclosing distance used
 # since anchor statistics are not readily accessible in MMDet
 enclose_cx = (enclose_x1 + enclose_x2) / 2
 enclose_cy = (enclose_y1 + enclose_y2) / 2
 rho_hat2 = (tgt_cx - enclose_cx) ** 2 + (tgt_cy - enclose_cy) ** 2
 # Normalize by squared diagonal of enclosing box
 r_hat = rho_hat2 / c2.clamp(min=eps) # [N], represents anchor quality
 r = rho2 / c2.clamp(min=eps) # [N], predicted center deviation

 # Focusing coefficient beta (WIoU v3 formula)
 # beta=1: degenerates to DIoU; beta>1: focuses on hard samples
 beta = torch.exp(r / (r_hat + eps)).detach() # detach: beta only provides gradient direction

 # ── WIoU Loss ─────────────────────────────────────────────────────────────
 # L_WIoU = beta * (1 - IoU + rho^2/c^2)
 wiou_loss = beta * (1 - iou + rho2 / c2.clamp(min=eps)) # [N]

 return wiou_loss.clamp(max=2.0)


@MODELS.register_module()
class WIoULoss(nn.Module):
 """WIoU v3 Loss for small object detection.

 Usage: directly replace the IoU Loss in YOLO/DETR detection heads;
 no other code changes required.

 Args:
 reduction (str): 'none' | 'mean' | 'sum'. Default 'mean'.
 loss_weight (float): Loss weight, corresponding to lambda_loc. Default 2.0.
 eps (float): Numerical stability constant. Default 1e-7.
 """

 def __init__(
 self,
 reduction: str = 'mean',
 loss_weight: float = 2.0,
 eps: float = 1e-7,
 ):
 super().__init__()
 self.reduction = reduction
 self.loss_weight = loss_weight
 self.eps = eps

 def forward(
 self,
 pred: torch.Tensor,
 target: torch.Tensor,
 weight: torch.Tensor = None,
 avg_factor: float = None,
 reduction_override: str = None,
 ) -> torch.Tensor:
 """Compute WIoU Loss.

 Args:
 pred (Tensor): Predicted boxes [N, 4], (x1, y1, x2, y2).
 target (Tensor): Target boxes [N, 4], (x1, y1, x2, y2).
 weight (Tensor): Sample weights [N].
 avg_factor (float): Average factor for normalization.
 reduction_override (str): Temporarily override the reduction method.

 Returns:
 Tensor: WIoU loss (scalar).
 """
 assert reduction_override in (None, 'none', 'mean', 'sum')
 reduction = reduction_override if reduction_override else self.reduction

 loss = bbox_overlaps_wiou(pred, target, eps=self.eps) # [N]

 if weight is not None:
 loss = loss * weight

 if avg_factor is not None:
 loss = loss.sum() / avg_factor
 elif reduction == 'mean':
 loss = loss.mean()
 elif reduction == 'sum':
 loss = loss.sum()

 return self.loss_weight * loss

 def extra_repr(self) -> str:
 return f'reduction={self.reduction}, loss_weight={self.loss_weight}'
