# Supplementary material for: DSPE-ViT: a lightweight vision transformer with dynamic sparse positional encoding for dense small object detection in UAV imagery
Source: Front Neurorobot. 2026 Jun 16;20:1849093. doi: 10.3389/fnbot.2026.1849093 (PMC13316741; doi:10.3389/fnbot.2026.1849093)
Supplement: Supplementary file 2 [file Supplementary_file_2.docx]

# Algorithms 1–3 (Section 3.2, transcribed)

The three algorithms below are the exact specifications cited at the end of Section 3.2 of the manuscript. Notation has been kept faithful to the paper; where the paper compresses a step into one line of prose, this transcript expands it into an explicit pseudocode step so a third party can re-implement without ambiguity.

Numbering convention (matches the manuscript):

- **Algorithm 1** — DSPE forward pass within a Transformer block (the integration view)
- **Algorithm 2** — PE Redundancy Pruner (sub-module of DSPE, Section 3.2.1)
- **Algorithm 3** — Local PE Enhancer with density-adaptive bias selection (sub-module of DSPE, Section 3.2.2)

*Notation. B = batch size, N = number of tokens (patches), D = embedding dimension, H = number of attention heads, W = window radius for the relative-position-bias table (so a (2W−1)² × H lookup), σ = sigmoid, ⊙ = element-wise product, 𝟙[·] = indicator (hard mask at inference).*

## Algorithm 1 — DSPE forward pass within a Transformer block

**Input.**

- Token features F ∈ ℝ^{B×N×D}
- Learnable absolute positional encoding P ∈ ℝ^{N×D}
- Learnable gate logits s ∈ ℝ^D (one per PE dimension)
- Density-conditional relative-position bias tables B_high, B_low ∈ ℝ^{(2W−1)²×H}
- Temperature τ (default 0.1)
- Transition bandwidth Δρ (default 0.1)

**Output.** Positional-aware token features F'.

**Steps.**

1. g ← σ(s / τ) during training; g ← 𝟙[g > 0.5] at inference. *(gating mask)*
2. P_eff ← g ⊙ P, broadcast across the token dimension. *(pruned PE)*
3. ρ ← AvgPool₃ₓ₃(‖F‖₂ along D), then min–max normalised to [0, 1] over the patch grid. *(density heatmap)*
4. τ_d ← Percentile₅₀(ρ). *(median density threshold)*
5. For each token t with density ρ_t:

- B_t ← B_low when ρ_t ≤ τ_d − Δρ
- B_t ← B_high when ρ_t ≥ τ_d + Δρ
- otherwise α_t ← clip((ρ_t − τ_d + Δρ) / (2Δρ), 0, 1) and

B_t ← α_t · B_high + (1 − α_t) · B_low.

1. F' ← MultiHeadAttention(Q, K, V; bias = B_t), where Q, K, V are linear projections of F + P_eff.

Return F'.

## Algorithm 2 — PE Redundancy Pruner (PERedundancyPruner)

**Input.**

- Positional encoding P ∈ ℝ^{N×D}
- Learnable gate logits s ∈ ℝ^D
- Temperature τ, binarisation threshold θ (default 0.5)
- Target retention ratio r_keep (the paper reports ≈ 62 % retained, i.e. r_keep ≈ 0.62)
- Training flag is_train

**Output.** Pruned positional encoding P_eff and sparsity loss L_sparse.

**Steps.**

1. g_soft ← σ(s / τ).
2. If is_train: g ← g_soft; else g ← 𝟙[g_soft > θ].
3. P_eff ← g ⊙ P, broadcast across the token dimension N.
4. L_sparse ← |mean(g) − r_keep|, contributing additively to the total loss during training.

Return (P_eff, L_sparse).

*The sparsity regularisation weight λ_sparse = 0.01 (from Implementation Details); the final loss adds λ_sparse · L_sparse to the detection loss.*

## Algorithm 3 — Local PE Enhancer with density-adaptive bias selection

**Input.**

- Token features F ∈ ℝ^{B×N×D}
- Two relative-position-bias tables B_high, B_low ∈ ℝ^{(2W−1)²×H} (trained independently; gradients backpropagate only through tokens in the matching density region)
- Transition bandwidth Δρ

**Output.** Density-conditional relative position bias B to be added to the attention logits.

**Steps.**

1. ρ ← AvgPool₃ₓ₃(‖F‖₂), then min–max normalised to [0, 1] on the spatial grid.
2. τ_d ← Percentile₅₀(ρ). *(50th-percentile threshold — high/low region split)*
3. For each token t with density ρ_t:

α_t ← clip((ρ_t − τ_d + Δρ) / (2Δρ), 0, 1). *(soft blending weight)*

1. B_t ← α_t · B_high + (1 − α_t) · B_low, degenerating to B_low when ρ_t ≤ τ_d − Δρ and to B_high when ρ_t ≥ τ_d + Δρ.
2. The per-token bias B_t is added to the attention logits at the multi-head self-attention layer.

Return B.

*Boundary handling. The half-width Δρ is measured on the min–max-normalised density distribution (so ρ_t ∈ [0, 1]); the default Δρ = 0.1 corresponds to a transition zone covering 10 % of the normalised density range on either side of the median threshold τ_d. This soft blending eliminates the attention discontinuities that would otherwise arise from hard partitioning at region boundaries.*

## Cross-reference to Supplementary File S1

| **Algorithm** | **S1 file** | **Symbol(s) in code** |
| --- | --- | --- |
| Algorithm 1 | dspe_module/dspe_module.py | class DSPEModule(BaseModule) |
| Algorithm 2 | dspe_module/redundancy_pruner.py | class PERedundancyPruner |
| Algorithm 3 | dspe_module/local_pe_enhancer.py | class LocalPEEnhancer |

# Hyperparameter & Configuration Summary

Single-table reference for every numerical knob used in the paper, gathered across Section 3 (Method), Section 4.1 (Implementation Details), and the ablation tables. Cite the manuscript for source of truth; this table exists so a re-implementer does not need to scan the prose for scattered numbers.

## Backbone — DSPE-ViT-Tiny

| **Hyperparameter** | **Value** | **Source** |
| --- | --- | --- |
| Embedding dimension D | 192 | §3.1 Overall architecture |
| Number of Transformer blocks | 6 (halved from DeiT-Tiny's 12) | §3.1 |
| Number of attention heads H | 3 | §3.1 |
| Patch size / stride | 16 × 16, stride 16 | §3.1 |
| Pretraining | DeiT-Tiny on ImageNet-1K | §4.1 |

## DSPE module

| **Hyperparameter** | **Value** | **Source** |
| --- | --- | --- |
| Temperature τ (PE gate) | 0.1 | §4.1 + §3.2.1 |
| Binarisation threshold θ (inference) | 0.5 | §3.2.1 / Algo 2 |
| Target retention ratio r_keep | ≈ 0.62 (≈ 119 / 192 dims kept after convergence) | §3.2.1 |
| Sparsity loss weight λ_sparse | 0.01 | §4.1 |
| Density pooling kernel | 3 × 3 average pool | §3.2.2 / Algo 3 |
| Density threshold τ_d | 50th percentile of ρ per layer | §3.2.2 / Algo 3 |
| Transition bandwidth Δρ | 0.1 (on normalised density) | §3.2.2 |
| Relative-position-bias table shape | (2W − 1)² × H per density region (two tables) | §3.2.2 |

## SmallObjFPN neck

| **Hyperparameter** | **Value** | **Source** |
| --- | --- | --- |
| Pyramid levels | {P2, P3, P4, P5} | §3.3 |
| P2 stride | 4 | §3.3 |
| Stem before patch-embedding | two 3×3 convs, stride 2 each, 3 → 32 → 64 channels (~0.02 M params) | §3.3 |
| Channel attention | SE block, squeeze ratio r = 16 | §3.3 |
| Fusion conv | depthwise-separable replacing standard 1×1 (≈ 75 % param reduction) | §3.3 |

## Detection head and loss

| **Hyperparameter** | **Value** | **Source** |
| --- | --- | --- |
| Head | ATSS (dynamic positive assignment via mean + std IoU) | §3.4 / [26] |
| Classification loss | Focal loss | §3.4 / [33] |
| Regression loss | WIoU v3 with dynamic focusing coefficient β | §3.4 / [25] |
| Auxiliary loss | Centre-ness | §3.4 |
| Total loss | L_cls + L_reg + L_ctr + λ_sparse · L_sparse | §3.4 + §3.2.1 |

## Training schedule

| **Hyperparameter** | **Value** | **Source** |
| --- | --- | --- |
| Framework | MMDetection 3.x | §4.1 |
| Hardware | single RTX 3080, 16 GB | §4.1 |
| Batch size | 8 | §4.1 |
| Epochs | 300 | §4.1 |
| Optimiser | AdamW | §4.1 |
| Initial learning rate | 2 × 10⁻⁴ | §4.1 |
| Weight decay | 0.05 | §4.1 |
| Schedule | Cosine annealing | §4.1 |
| Warm-up | 5 epochs, linear | §4.1 |
| Input resolution | 640 × 640 | §4.1 |
| Augmentation | Mosaic + RandomFlip + AutoAugment | §4.1 |

## Datasets and evaluation

| **Item** | **Value** | **Source** |
| --- | --- | --- |
| Primary benchmark | VisDrone2019-DET — 10 classes, 10,209 images (train 6,471 / val 548 / test-dev 1,610 / test-challenge 1,580) | §4.0 |
| Image resolution | 960 × 540 to 2000 × 1500 | §4.0 |
| Small-object fraction | > 87 % (pixel width < 32 px, train-set statistics) | §4.0 |
| Validation evaluation set | val (548 images), unless otherwise noted | §4.0 |
| Test-dev re-evaluation | 1,610 images, identical protocol, Table 1-B | §4.2 |
| Cross-domain dataset | SeaDronesSee — 4 classes, 5,630 train / 859 val | §4.0 |
| Cross-domain protocols | zero-shot transfer + 50-epoch fine-tune (UAVDT) | §4.0 / §4.x |
| Metrics | mAP@0.5, mAP@0.5:0.95 | §4.0 |
| Efficiency metrics | Params (M), GFLOPs @ 640×640, FPS @ RTX 3080 batch 1 | §4.0 |

## Reported headline numbers (for sanity-check after re-implementation)

| **Surface** | **Metric** | **Value** | **Source** |
| --- | --- | --- | --- |
| VisDrone val (548 images) | mAP@0.5 | 43.2 % ± 0.3 (mean over 3 runs) | Table 1 |
| VisDrone val | mAP@0.5:0.95 | 24.1 % ± 0.2 | Table 1 |
| VisDrone val | Params / GFLOPs / FPS | 6.0 M / 15.8 / 64 | Table 1 |
| VisDrone test-dev (1,610) | mAP@0.5 / mAP@0.5:0.95 | 41.9 % / 23.2 % | Table 1-B |
| SeaDronesSee val | mAP@0.5 (zero-shot / fine-tuned) | 30.1 % / 38.4 % | Abstract / §4.x |
| UAVDT (cross-dataset) | mAP@0.5 (zero-shot / fine-tuned) | 27.9 % / 32.4 % | §4.x |

# Independent Re-implementation Guide

This guide is the third-party complement to the running code in Supplementary File S1. It walks through what a reader needs to do to rebuild DSPE-ViT in any deep-learning framework, not just MMDetection, and to reproduce the headline numbers reported in the paper (Tables 1 / 1-A / 1-B). All numerical values reference `hyperparameters.md`; all algorithmic specifications reference `algorithms.md`.

The text below is deliberately written at the level of "what an experienced detection-engineer would need to be told." It does not duplicate prose already in the manuscript; it converts that prose into an actionable plan.

## 1. Build the backbone (DSPE-ViT-Tiny)

Start from any DeiT-Tiny implementation that exposes per-block hooks before the multi-head self-attention layer. Three modifications are required:

1. **Halve the depth** from 12 to 6 Transformer blocks. Keep D = 192,

H = 3, patch size 16 with stride 16. Initialise from DeiT-Tiny ImageNet-1K weights — the first 6 blocks transfer one-to-one.

1. **Insert the DSPE module** before the MHSA call in every block.

Algorithm 1 in `algorithms.md` is the spec. The two sub-modules are Algorithm 2 (gating) and Algorithm 3 (density-adaptive relative bias). Wire the gating mask g to multiply the absolute PE *before* it is added to the token features; wire the bias B_t as an additive term *inside* the attention-logit matrix so it composes with the standard softmax(QKᵀ / √d) formulation.

1. **Add the convolutional stem for P2.** Two 3×3 stride-2 convs

(3 → 32 → 64), placed *before* the patch embedding, so that the stem's stride-4 feature map can be projected to FPN channels via a 1×1 conv and fused with P3 inside the neck. This costs ≈ 0.02 M params.

The output side of the backbone must expose feature maps at strides {8, 16, 32} (i.e. {P3, P4, P5} in FPN convention), plus the separately extracted stride-4 stem map for P2.

## 2. Build the neck (SmallObjFPN)

Standard top-down FPN with three deltas:

1. Add a **P2 level** (stride 4) by lateral-projecting the stem and fusing

with P3 via the usual top-down + nearest-neighbour upsample pattern.

1. Replace the 1×1 fusion convs by **depthwise-separable convs**

(depthwise + pointwise + BN + activation). Roughly 75 % fewer params per fusion path than the standard FPN.

1. Apply **SE channel attention** (squeeze ratio r = 16) to each output

level after fusion. Implementation: GAP → FC(C → C/16) → ReLU → FC(C/16 → C) → Sigmoid → channel-wise multiplication.

Output a four-level pyramid {P2, P3, P4, P5} to the head.

## 3. Build the detection head and loss

- **Head:** ATSS [26] — dynamic positive-sample assignment using

mean(IoU) + std(IoU) between candidate anchors and ground-truth centres as the per-image threshold. Anchor-free single-anchor-per-level is fine if the implementation matches the original ATSS protocol.

- **Classification:** Focal loss [33], standard γ = 2, α = 0.25.
- **Regression:** WIoU v3 [25] — substitute 1 − WIoU(b̂, b) for the

usual IoU/GIoU/CIoU loss. The dynamic focusing coefficient β is the point of WIoU v3 and is automatically derived from the geometric out-of-distribution ratio; consult the WIoU paper or the wiou_loss.py in S1 for the exact closed form. Do not omit the dynamic-focusing branch — Section 5 of the paper attributes a measurable share of the small-object gain to it.

- **Centre-ness:** standard centre-ness auxiliary loss as in FCOS / ATSS.
- **Sparsity:** add λ_sparse · L_sparse (with L_sparse from

Algorithm 2) to the total. Default λ_sparse = 0.01.

Total objective: L = L_focal + L_wiou + L_ctr + λ_sparse · L_sparse.

## 4. Datasets

### VisDrone2019-DET (primary)

Download from https://github.com/VisDrone/VisDrone-Dataset. The official splits are 6,471 train / 548 val / 1,610 test-dev / 1,580 test-challenge. Convert annotations to MS-COCO format (bbox = [x, y, w, h] in pixels). Train on the official train split; report on the **validation set (548 images)** for Table 1 and on the **test-dev set (1,610 images)** for Table 1-B. Do *not* train on the val set.

### SeaDronesSee (cross-domain)

Download from https://seadronessee.cs.uni-tuebingen.de/. Four classes, 5,630 train / 859 val. Used for two protocols:

- **Zero-shot:** apply the VisDrone-trained model directly to SeaDronesSee

val. Map class labels through the obvious correspondences; report mAP@0.5 only.

- **Fine-tune:** continue training for 50 epochs on the SeaDronesSee

train split with the same optimiser/schedule, learning rate scaled to 1 × 10⁻⁴.

### UAVDT (additional cross-dataset check)

Used only as a robustness probe; same two protocols as SeaDronesSee. Targets in the paper: 27.9 % mAP@0.5 zero-shot, 32.4 % after 50-epoch fine-tune.

## 5. Training schedule

Single RTX 3080 (16 GB), batch size 8. 300 epochs, AdamW with lr = 2e-4, weight_decay = 0.05, cosine annealing, 5-epoch linear warm-up. Input 640 × 640, augmentations: Mosaic + RandomFlip + AutoAugment. See `hyperparameters.md` for the complete numerical table.

## 6. Evaluation

- Metrics: mAP@0.5, mAP@0.5:0.95, Params (M), GFLOPs at 640×640

input, and FPS at 640×640 batch 1 on an RTX 3080.

- For statistical reliability the paper reports DSPE-ViT as

mean ± standard-deviation over **three** independent training runs with different seeds; expect ≈ ±0.3 mAP@0.5 spread.

- For per-class analysis (Table 1-A), compute AP@0.5 per VisDrone

category and verify the inverse correlation between target scale and improvement magnitude described in §4.2.

## 7. Sanity checks for a third-party re-implementation

Use these as smoke tests once training converges; numbers come from the manuscript and are reproduced in `hyperparameters.md`:

| **Check** | **Expected** | **Tolerance to flag a bug** |
| --- | --- | --- |
| Param count of full model | 6.0 M | > 6.5 M means the depth halving or depthwise-separable fusion was missed |
| GFLOPs at 640×640 | 15.8 | > 18 means the standard FPN was used instead of SmallObjFPN |
| Average PE-gate mean(g) at convergence | ≈ 0.62 | < 0.4 means λ_sparse is too high; > 0.85 means it is too low |
| VisDrone val mAP@0.5 | 43.2 ± 0.3 | < 41 means the DSPE module, the P2 branch, or WIoU v3 is mis-wired |
| SeaDronesSee val mAP@0.5, zero-shot | 30.1 | < 25 indicates the VisDrone training already over-specialised on urban scenes |

If multiple checks fail simultaneously, inspect (in this order) the gating mask broadcasting in step 2 of Algorithm 1, the density normalisation in step 3, and the bias-table backpropagation masking described under Algorithm 3 (gradients must only flow through the matching density region).

## 8. What this guide does *not* cover

Deployment-side TensorRT INT8 quantisation, profiling on edge hardware, and the Grad-CAM visualisation pipeline are described in Section 4 and Section 5 of the manuscript and are out of scope for re-implementing the *model*. The full deployment scripts are part of the GitHub release: https://github.com/LiyaCai-001/DSPE-ViT-1.
